# The LARGE1 controls grain size by repressing the interaction between PGL2 and APG in rice

**DOI:** 10.1111/tpj.70674

**Published:** 2026-01-16

**Authors:** Yapei Liu, Hao Zhang, Ying Gao, Xingni Xi, Yuhan Zhang, Jia Lyu, Limin Zhang, Yunhai Li

**Affiliations:** ^1^ State Key Laboratory of Seed Innovation Institute of Genetics and Developmental Biology, Chinese Academy of Sciences Beijing 100101 China; ^2^ State Key Laboratory of Crop Gene Resources and Breeding/National Key Facility for Crop Gene Resources and Genetic Improvement Institute of Crop Sciences, Chinese Academy of Agricultural Sciences Beijing 100081 China; ^3^ Key Laboratory of Forage and Endemic Crop Biotechnology, School of Life Sciences Inner Mongolia University Hohhot China; ^4^ University of Chinese Academy of Sciences Beijing 100039 China

**Keywords:** *Oryza sativa*, grain size, LARGE1, GSK2, PGL2, APG

## Abstract

Grain size has long been recognized as a key determinant of yield potential in crops. Understanding the mechanisms governing grain size is critical for breeding high‐yielding varieties. In a previous work, we revealed that the RNA‐binding protein LARGE1 acts as a negative regulator of grain size and weight in rice. LARGE1 interacts with GSK2 (GLYCOGEN SYNTHASE KINASE2) and is phosphorylated by GSK2. Here, we report that LARGE1 physically interacts with an atypical non‐DNA‐binding bHLH protein PGL2 that positively influences grain size. Biochemical analyses show that PGL2 binds to APG, a typical DNA‐binding bHLH protein that negatively regulates grain size. PGL2 suppresses the transcriptional activation activity of APG by forming the PGL2/APG heterodimer. Strikingly, LARGE1 can repress the formation of the heterodimer PGL2/APG by competitively binding PGL2, thereby releasing the inhibitory effect of PGL2 on the transcriptional activation activity of APG. Genetic evidence and RNA‐seq analyses support that *LARGE1* and *PGL*2 act in a common pathway to regulate grain size in rice. Our findings uncover a novel regulatory module GSK2‐LARGE1‐PGL2/APG that fine‐tunes grain size, suggesting a promising target for improving seed size and weight in crops.

## Significance Statement

Grain size is a key agronomic trait for improving cereal yield. The RNA‐binding protein LARGE1 limits rice grain size by directly competing with the transcription factor APG for binding to the positive regulator PGL2. This work elucidates a complete signaling module GSK2‐LARGE1‐PGL2/APG that fine‐tunes grain size, highlighting the novel link between RNA‐binding protein and transcription factors as a promising target for breeding.

## INTRODUCTION

To address the food demands of the rapidly growing global population, global grain production must increase by over 50% by 2050 (Bailey‐Serres et al., 2019; Yu et al., 2021). Rice (*Oryza sativa* L.) is one of the most important food crops worldwide, consumed by over half of the global population (Liu et al., 2014). Therefore, breeding high‐yielding rice varieties is crucial for ensuring food security. Grain size, which is determined primarily by grain length, width, and thickness, is a key determinant of rice yield (Li et al., 2019). Multiple signaling pathways, such as the ubiquitin‐proteasome pathway, G‐protein signaling, MAPK signaling, phytohormone pathways, and transcriptional regulators, have been identified to regulate grain size (Li et al., 2019).

Transcriptional regulation plays a critical role in plant growth and developmental processes in plants. Multiple transcription factors, such as GRFs, bHLHs, SPLs, WRKYs, and AP2 family members, have been implicated in controlling grain size (Che et al., 2015; Duan et al., 2015; Hu et al., 2015; Koichiro et al., 2014; Li et al., 2016; Si et al., 2016; Sun et al., 2016; Tian et al., 2017; Wang et al., 2012; Yu et al., 2017; Yuan et al., 2019; Zhang et al., 2021). Basic helix–loop–helix (bHLH) transcription factors are known to mediate brassinosteroid (BR) signaling to regulate cell elongation. Based on DNA‐binding activity, bHLH proteins are categorized into two groups, atypical non‐DNA‐binding HLH proteins and typical DNA‐binding bHLH proteins (Li et al., 2006). These proteins can act as either positive or negative regulators of growth, interacting antagonistically and redundantly to modulate cell elongation. In Arabidopsis, studies have characterized an antagonistic bHLH regulatory system that elucidates the functional relationship between bHLH and atypical HLH proteins in modulating cell elongation (Ikeda et al., 2012). A representative example involves PACLOBUTRAZOL RESISTANCE1 (PRE1) and its interactors. The PRE family proteins, which lack the DNA‐binding basic domain characteristic of typical bHLH factors, positively regulate cell elongation through competitive inhibition of atypical bHLH repressors such as INCREASED LEAF INCLINATION1 BINDING bHLH1 (IBH1) and ACTIVATION TAGGED BRI1 SUPPRESSOR1 INTERACTING FACTORs (AIFs) (Bai et al., 2012; Fan et al., 2014; Hyun & Lee, 2006; Ikeda et al., 2012; Wang et al., 2009; Zhang et al., 2009). In rice, functional homologs of this regulatory network have been identified. BRASSINOSTEROID UPREGULATED1 (BU1, OsbHLH172), showing high sequence similarity to Arabidopsis PRE1, positively regulates BR signaling (Tanaka et al., 2009). Conversely, OsIBH1 acts as a negative regulator of cell elongation by interacting with positive regulators OsBC1‐LIKE proteins OsBCL1 (OsbHLH80) and OsBCL2 (OsBLR1/OsbHLH79). These interactions modulate BR‐mediated cell expansion processes in laminar joints and grain development (Jang et al., 2021; Seo et al., 2020). Furthermore, ILI1, which binds OsIBH1 along with OsbHLH157 and OsbHLH158, was identified as the negative atypical bHLH factors to regulating cell elongation in rice (Zhang et al., 2009). Notably, a tripartite antagonistic cascade involving two atypical HLH proteins (OsAIF1/OsbHLH176 and OsAIF2/OsbHLH178) and the bHLH factor OsbHLH92 has been identified (Lu et al., 2025). This regulatory module dynamically balances leaf angle determination and grain size control through BR signaling pathways, with the atypical HLHs acting as negative regulators that antagonize OsbHLH92's activity (Lu et al., 2025). Additionally, the non‐DNA‐binding bHLH factors PGL1 (POSITIVE REGULATOR OF GRAIN LENGTH 1) or PGL2 (POSITIVE REGULATOR OF GRAIN LENGTH 2, also known as OsBUL1 or OsbHLH170) form inhibitory heterodimers with the canonical bHLH transcription factor APG (ANTAGONIST OF PGL1/OsPIL16), respectively, thereby suppressing APG‐mediated transcriptional regulation in grain development (Heang & Sassa, 2012a, 2012b). Consistent with this regulatory mechanism, both PGL1 and PGL2 positively regulate grain length by promoting cell expansion in the spikelet hull, whereas APG exerts a negative influence on rice grain length (Heang & Sassa, 2012c). PGL2/OsBUL1 modulates rice leaf angle and grain size through an HLH‐bHLH transcriptional regulatory complex composed of PGL2/OsBUL1, LO9‐177 and OsBC1 (Jang et al., 2017). The regulatory network involves an additional two bHLH factors, OsbHLH98, and counteracts BR‐induced cell elongation by transcriptionally repressing PGL2/OsBUL1 (Guo et al., 2021), while OsbHLH92 directly activates the transcription of both *OsBU1* and *PGL2/OsBUL1* by binding their promoters (Teng et al., 2023). Although these findings establish crucial roles for bHLH proteins in BR‐induced cell elongation, it is still unclear whether and how other factors regulate the HLH‐bHLH transcriptional regulatory complex to influence grain size.

We previously demonstrated that LARGE1, a Mei2‐like protein 4 (OML4) with three RNA recognition motif (RRM) domains, regulates grain size by restricting cell expansion within spikelet hulls in rice. This stabilization of LARGE1 is mediated through its interaction with and phosphorylation by GSK2 (Lyu et al., 2020). It is well‐established that RNA‐binding proteins (RBPs) exert critical regulatory functions in post‐transcriptional processes, including RNA splicing, modification, transport, subcellular localization, stability control, translation regulation, and degradation (Holmqvist & Vogel, 2018; Van Nostrand et al., 2020; Wang, Arai, et al., 2008). However, their roles in transcriptional regulation remain relatively underexplored. Previous studies have demonstrated that approximately 60% of human RNA‐binding proteins exhibit extensive chromatin interactions, with significant enrichment observed at gene promoters and enhancer regions. Specifically, experimental evidence confirms that RBM25 modulates chromatin architecture and transcriptional activation/repression by regulating the association of RNA‐dependent transcription factor YY1 with chromatin (Xiao et al., 2019). Notably, the discovery of RBPs competitively interacting with transcription factors directly to regulate their transcriptional activity represents a novel regulatory mechanism not previously documented. In this study, we identify a critical regulatory cascade, LARGE1‐PGL2‐APG, that modulates grain size in rice. We show that LARGE1 directly interacts with PGL2 and competitively disrupts the binding of PGL2 to APG, thereby governing grain size. Genetic analyses and RNA‐seq data further confirm that *LARGE1* and *PGL2* act in a common pathway to regulate grain size in rice. Thus, our findings reveal a previously unknown molecular mechanism that LARGE1 antagonizes PGL2 to modulate the transcriptional activation activity of APG, ultimately controlling grain size in rice.

## RESULTS

### LARGE1 physically interacts with PGL2

**Figure 1:**
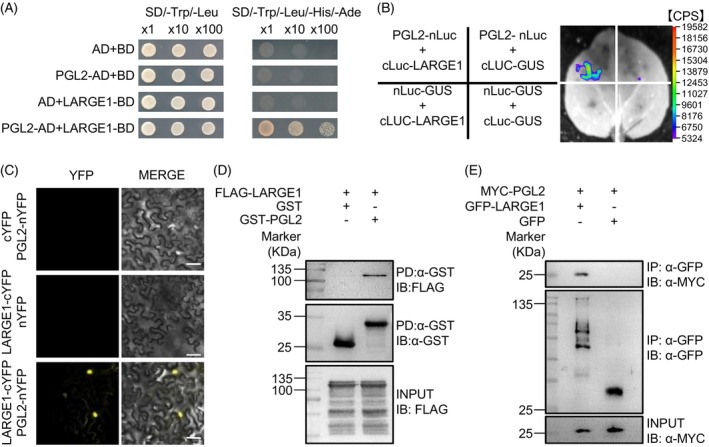
LARGE1 physically interacts with PGL2 *in vitro* and *in vivo*. (A) LARGE1 interacts with PGL2 in yeast cells cultured on SD/−Trp/−Leu or SD/−Trp/−Leu/−His/−Ade media. (B) LARGE1 associates with PGL2 in *Nicotiana benthamiana* leaves using a split‐luciferase complementation assay. Co‐expression of PGL2‐nLUC and cLUC‐LARGE1 constructs via *Agrobacterium*‐mediated infiltration resulted in detectable luciferase activity 48 h post‐infiltration. Luminescence intensity, represented by a pseudocolor scale, confirmed the interaction. (C) Bimolecular fluorescence complementation (BiFC) analysis demonstrated the interaction between LARGE1 and PGL2 in *N. benthamiana* leaves. Fluorescent signal reconstitution was observed following co‐expression of C‐terminal YFP‐tagged LARGE1 (LARGE1‐cYFP) and N‐terminal YFP‐fused PGL2 (PGL2‐nYFP) in leaf epidermal cells. (D) *In vitro* pull‐down assay demonstrating LARGE1‐PGL2 physical interaction. Recombinant FLAG‐tagged LARGE1 protein was incubated with GST‐fused PGL2, followed by affinity isolation using anti‐FLAG magnetic beads. Protein complexes were subsequently analyzed by immunoblotting (IB) with GST‐specific antibodies. IB, immunoblot. (E) Protein association of LARGE1 with PGL2 demonstrated by co‐immunoprecipitation analysis. Protein complexes were isolated from two transgenic rice lines: *pro35S:MYC‐PGL2; pro35S:GFP‐LARGE1* (test) and *pro35S:MYC‐PGL2; pro35S:GFP* (control). GFP‐trap beads were employed for protein complex isolation followed by immunoblot detection using anti‐MYC and anti‐GFP monoclonal antibodies. Scale bar represents 50 μm in panel (C).

To further investigate the role of LARGE1 in regulating rice grain size, we performed a yeast two‐hybrid (Y2H) screen to identify its interacting proteins. Using the full‐length LARGE1 protein as the bait, we identified PGL2 as one of the interaction partners. PGL2 was previously reported to positively regulate grain size by promoting cell elongation (Heang & Sassa, 2012b), suggesting that PGL2 may function with LARGE1 to control rice grain size. Subsequently, we first confirmed that LARGE1 can interact with the full‐length PGL2 in yeast cells (Figure 1A). To determine whether LARGE1 can directly interact with PGL2, we conducted an *in vitro* pull‐down assay. FLAG‐tagged LARGE1 (FLAG‐LARGE1) and GST‐tagged PGL2 (GST‐PGL2) proteins were expressed in *E. coli*. As shown in Figure 1(D), FLAG‐LARGE1 specifically binds to GST‐PGL2, but not to the GST control, indicating that LARGE1 directly interacts with PGL2 *in vitro*.

To validate the interaction between LARGE1 and PGL2 *in vivo*, we employed two complementary approaches. First, a luciferase complementation imaging (LCI) assay was performed. PGL2 and LARGE1 were fused to the N‐terminal (nLUC) and C‐terminal (cLUC) fragments of luciferase for generating PGL2‐nLUC and cLUC‐LARGE1, respectively. Subsequently, transient co‐expression of PGL2‐nLUC and cLUC‐LARGE1 in *Nicotiana benthamiana* leaves resulted in detectable luciferase activity (Figure 1B), whereas negative controls showed no signal. Second, a bimolecular fluorescence complementation (BiFC) assay was conducted. PGL2 and LARGE1 were fused to the N‐ and C‐terminal halves of yellow fluorescent protein (YFP), respectively. Co‐expression of PGL2‐nYFP and LARGE1‐cYFP in *Nicotiana benthamiana* leaves restored YFP fluorescence, which was localized predominantly to the nuclei of epidermal cells (Figure 1C). These results further corroborated the interaction between LARGE1 and PGL2 *in vivo*. Finally, to examine the association of LARGE1 with PGL2 in rice, we further performed co‐immunoprecipitation (Co‐IP) assays using transgenic plants co‐expressing epitope‐tagged LARGE1 (35S::GFP‐LARGE1) and PGL2 (35S::MYC‐PGL2). Co‐IP analyses demonstrated that MYC‐PGL2 physically associates with GFP‐LARGE1, but not with the GFP control (Figure 1E). Thus, these results establish that LARGE1 physically interacts with PGL2 both *in vitro* and *in vivo*.

### LARGE1 competitively inhibits the interaction between PGL2 and APG

**Figure 2:**
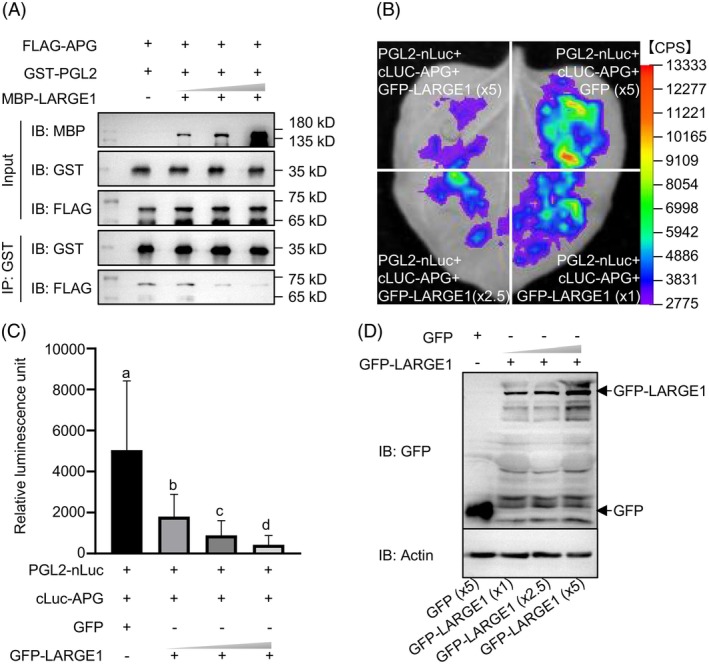
LARGE1 competitively inhibits the interaction between PGL2 and APG. (A) *In vitro* pull‐down assay demonstrates LARGE1‐mediated inhibition of APG‐PGL2 interaction. FLAG‐tagged APG proteins demonstrated concentration‐dependent reduction in co‐immunoprecipitation efficiency with GST‐PGL2 upon titration of MBP‐LARGE1 fusion proteins. Immunoblots depict: (1) Input controls verifying equivalent loading of recombinant proteins; (2) GST pull‐down complexes showing specific interaction profiles; (3) Attenuation of FLAG‐APG/GST‐PGL2 complex formation proportional to MBP‐LARGE1 concentration gradient. (B) The Split‐luciferase complementation assay reveals GFP‐LARGE1‐mediated inhibition of PGL2‐nLUC/cLUC‐APG complex formation. Transient co‐expression assays were performed by infiltrating *Nicotiana benthamiana* leaves with *Agrobacterium* cultures carrying 35S promoter‐driven constructs (*PGL2‐nLUC*, *cLUC‐APG*, *GFP*, or *GFP‐LARGE1*) in designated combinations. Luminescence signals were detected using d‐luciferin as a luciferase substrate. (C) Luminescent intensities shown in (B) were quantitatively assessed and statistically evaluated. Values represent mean ± SD (*n* = 48). Lowercase letters above bars denote significant differences (one‐way ANOVA with *post hoc* test, *P* < 0.01). (D) Immunoblot analysis comparing GFP and GFP‐LARGE1 expression profiles across experimental groups in (B). Protein lysates were immunoprobed using epitope‐specific antibodies against GFP (target analyte) and Actin (endogenous control).

Previous studies demonstrated that PGL2, an atypical bHLH transcription factor lacking a DNA‐binding domain, interacts with the typical bHLH transcription factor APG, a negative regulator of grain size in rice (Heang & Sassa, 2012a, 2012b, 2012c). It is well‐established that HLH and bHLH transcription factors form inactive heterodimers, thereby inhibiting the DNA‐binding capacity of bHLH transcription factors (Ellis, 1994; Sun et al., 1991). To investigate whether LARGE1 modulates the formation of the PGL2/APG heterodimer, we conducted a pull‐down assay using tagged fusion proteins expressed in *Escherichia coli*. FLAG‐tagged APG (FLAG‐APG), GST‐tagged PGL2 (GST‐PGL2), and MBP‐tagged LARGE1 (MBP‐LARGE1) were individually expressed in *E. coli*. When equal amounts of FLAG‐APG and GST‐PGL2 were incubated with anti‐GST beads, FLAG‐APG co‐purified with GST‐PGL2. However, incremental addition of MBP‐LARGE1 to the mixture progressively reduced FLAG‐APG co‐purification with GST‐PGL2 in a concentration‐dependent manner (Figure 2A). These results indicated that LARGE1 competitively disrupts the PGL2‐APG interaction by binding to PGL2. We next investigated whether LARGE1 could also interact directly with APG to affect the PGL2‐APG complex. To address this, we performed an *in vitro* pull‐down assay. The pull‐down assay failed to detect a physical interaction (Figure S4). Thus, the competitive inhibition of the PGL2‐APG interaction by LARGE1 is more likely attributed to its interaction with PGL2.

To further verify whether LARGE1 competes with APG for binding PGL2 in plant cells, we performed a luciferase complementation imaging (LCI) assay. *Agrobacterium* strains expressing PGL2‐nLUC and cLUC‐APG were co‐infiltrated into *Nicotiana benthamiana* leaves with strains expressing GFP (negative control) or increasing concentrations of GFP‐LARGE1. As shown in Figure 2(D) and Figure S6, the protein levels of GFP‐LARGE1 increased proportionally with the concentration of the corresponding *Agrobacterium* strain. Conversely, the firefly luciferase signals diminished as GFP‐LARGE1 levels increased (Figure 2B,C), indicating that GFP‐LARGE1 specifically inhibits PGL2–APG interaction, whereas GFP does not. Together, these findings further confirmed that LARGE1 competitively represses the interaction between PGL2 and APG. Furthermore, we verified that the LARGE1–PGL2 interaction does not alter the protein stability and subcellular localization of either PGL2 or APG (Figures S5 and S6).

### LARGE1 alleviates the inhibition of PGL2 on APG transcriptional activation activity

**Figure 3:**
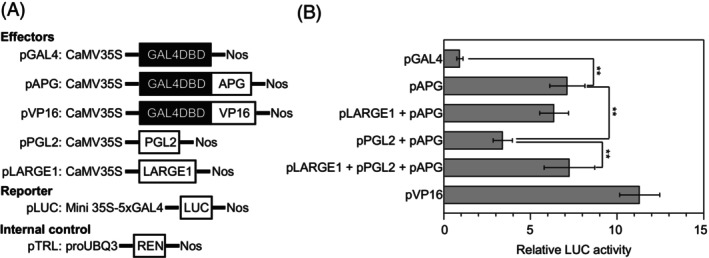
LARGE1 relieves PGL2‐mediated suppression of APG transcriptional activation activity. (A) In the dual‐luciferase reporter (DLR) assay system, the effector, reporter, and internal control constructs are as follows: The reporter gene firefly luciferase (LUC) and the internal control Renilla luciferase (REN) are cloned into separate plasmids. For transient transfection assays, these constructs are co‐transfected at a 6:1 ratio (reporter:internal control) to ensure normalized quantification. (B) Transcriptional activation ability of APG was quantified through normalized relative luciferase activity measurements using the reporter systems illustrated in (A). Effector constructs pGAL4 and pVP16 were used as negative and positive controls, respectively. Values are given as means ± SD (*n* = 10). GAL4DBD is short for GAL4 DNA‐binding domain. 5 × GAL4, five copies of the GAL4 binding element. LUC, the firefly luciferase gene; REN, the Renilla luciferase gene. Statistical significance thresholds: **P < 0.01 (two‐tailed Student's *t*‐test).

Atypical bHLH proteins typically function as inhibitors of typical bHLH proteins via dimerization (Toledo‐Ortiz et al., 2003). For instance, IBH1, a transcriptional repressor lacking DNA‐binding ability, forms heterodimers with HBI1 and ACE1, thereby suppressing their transcriptional activities and leading to growth retardation (Bai et al., 2012; Fan et al., 2014; Ikeda et al., 2012). Similarly, APG, a bHLH transcription factor, interacts with the atypical bHLH protein PGL2 to form an HLH‐bHLH heterodimer (Figure 2A; Figure S2) (Heang & Sassa, 2012b). To determine whether the interaction between LARGE1 and PGL2 influences APG's transcriptional activation activity, we employed a transient dual‐luciferase reporter assay in rice protoplasts (Hao et al., 2011). This system is employed to assess the effects of PGL2, LARGE1, and their combinatorial interactions on the transcriptional activity of APG. Effector constructs pGAL4 and pVP16 served as negative and positive controls, respectively (Figure 3). Compared to the negative control, APG demonstrated robust activation of the LUC reporter gene, resulting in a significant increase in relative luciferase activity. Co‐expression of APG with LARGE1 had minimal impact on luciferase activity. However, co‐expression of APG with PGL2 markedly reduced transcriptional activity relative to APG alone (Figure 3B), indicating that PGL2 negatively regulates the transcriptional activity of APG. Strikingly, when LARGE1 was co‐expressed with both PGL2 and APG, luciferase activity recovered to levels comparable to APG expression alone (Figure 3B). We confirmed this same regulatory trend in tobacco leaf epidermal cells using the dual‐luciferase reporter (DLR) assay (Figure S7). Collectively, these results demonstrated that LARGE1 can alleviate the inhibition of PGL2 on APG transcriptional activation activity.

### *PGL2* acts genetically with *LARGE1* to regulate grain size

Considering that LARGE1 interacts with PGL2 and modulates its inhibitory effect on the transcriptional activation activity of APG, we investigated whether LARGE1 and PGL2 have overlapped roles in grain size regulation. Although PGL2 has been implicated in rice grain length control (Heang & Sassa, 2012b; Jang et al., 2017), its detailed function in grain size remains unclear. To elucidate the genetic relationship between LARGE1 and PGL2, we generated two loss‐of‐function mutants of *PGL2* (*pgl2‐cri*) using the CRISPR/Cas9 method (Figure S1a,b). The *pgl2‐cri* mutants exhibited significantly shorter and narrower grains compared to wild type ZH11 (Figure S1d,g,h). Conversely, *PGL2* overexpression lines (*PGL2‐OE*) developed longer and wider grains (Figure S3), demonstrating that PGL2 positively regulates both grain length and width. In contrast, a previous study showed that the T‐DNA insertion mutant *Osbul1/pgl2* displayed only reduced grain length (Jang et al., 2017). This discrepancy may arise from allelic differences or distinct genetic backgrounds. Additionally, *pgl2‐cri* mutants showed a reduction in grain weight (Figure S1m), short panicle length (Figure S1e,k), and increased primary panicle branch number (Figure S1i). However, secondary panicle branches and grain number per panicle remained unchanged (Figure S1j,l). The *PGL2‐OE* lines exhibited increased grain size and plant height, but no significant changes in panicle architecture (Figure S3).

**Figure 4:**
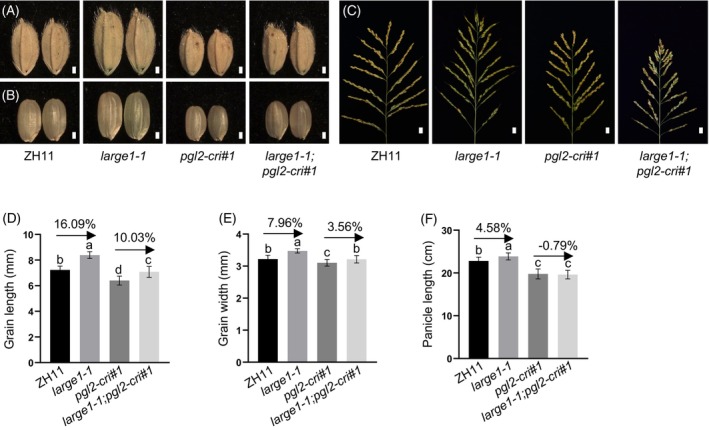
*PGL2* acts genetically with *LARGE1* in rice. (A, B) Grain morphology of wild type ZH11, *large1‐1*, *pgl2‐cri#1*, and *large1‐1;pgl2‐cri#1* mutants. Scale bar: 1 mm. (C) Panicle morphology of the indicated genotypes. Scale bar: 1 cm. (D, E) Grain length and grain width measurements. (F) Panicle length analysis. Data in (D–F) represent means ± SD [*n* ≥ 82 for (D, E); *n* ≥ 14 for (F)]. Lowercase letters above columns denote statistically significant differences (*P* < 0.01, one‐way ANOVA with Tukey's *post hoc* test).

To further explore the genetic relationship between *LARGE1* and *PGL2*, we generated *large1‐1;pgl2‐cri#1* double mutant by crossing *large1‐1* with *pgl2‐cri#1* (Figure 4A–D). Compared to wild type ZH11, the *large1‐1* single mutant increased grain length by 16.09% and grain width by 7.96%. However, when compared to *pgl2‐cri#1*, the *large1‐1;pgl2‐cri#1* double mutant exhibited only a 10.03% increase in grain length and a 3.56% increase in grain width (Figure 4E,F). Furthermore, the panicle length of the double mutant *large1‐1;pgl2‐cri#1* resembled that of *pgl2‐cri#1* (Figure 4G). These results indicate that *pgl2‐cri#1* partially suppresses the enhanced grain size phenotypes of *large1‐1*, suggesting that *PGL2* and *LARGE1* function, at least in part, in a genetic pathway to regulate grain length and width in rice.

Given that GSK2‐mediated phosphorylation regulates LARGE1 protein levels, we asked whether *GSK2* and *PGL2* could function in a common pathway to control grain size. To test this, we generated *GSK2‐RNAi;pgl2‐cri#1* double mutants (Figure S11). The *GSK2‐RNAi* lines alone increased grain length by 11.56% compared to the wild type (ZH11). Notably, the *GSK2‐RNAi;pgl2‐cri#1* double mutants exhibited a 9.53% increase in grain length relative to the *pgl2‐cri#1* single mutants (Figure S11c). The grain width of *GSK2‐RNAi;pgl2‐cri#1* was similar to that of the *pgl2‐cri#1* single mutant (Figure S11d). Collectively, these results support that *GSK2* acts genetically with *PGL2* to regulate grain size.

### *LARGE1* and *PGL2* possess overlapping downstream genes

**Figure 5:**
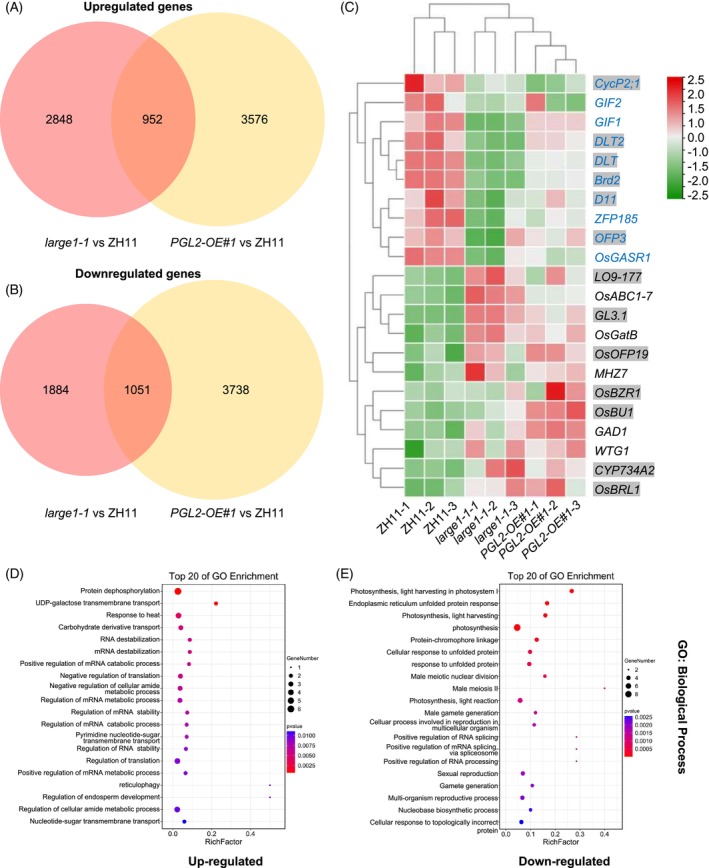
Comparative analysis of transcriptional alterations in *large1‐1* and *PGL2‐OE#1* plants. (A, B) Venn diagram displaying differentially expressed genes in *large1‐1* and *PGL2‐OE#1*. (A) Upregulated genes in *large1‐1* and *PGL2‐OE#1* were 3800 and 4528, respectively, in which 952 genes were upregulated in both *large1‐1* and *PGL2‐OE#1*. (B) Downregulated genes in *large1‐1* and *PGL2‐OE#1* were 2935 and 4789, respectively, in which 1051 genes were downregulated in both *large1‐1* and *PGL2‐OE#1*. (C) Clustered heat map depicting 22 overlapping genes associated with rice grain size. Gene expression levels are visualized across samples, color‐coded from bright green (lowest expression) to bright red (highest expression). Upregulated genes are labeled in black text, and downregulated genes in blue text. Thirteen genes are highlighted to denote their roles in brassinosteroid (BR) synthesis or signaling pathways. (D, E) Top 20 enriched Gene Ontology (GO) terms in the Biological Process category for (D) upregulated and (E) downregulated differentially expressed genes (DEGs) shared between the *large1‐1* and *PGL2‐OE#1* lines. Dot size reflects the number of genes per term, and color intensity indicates statistical significance [−log_10_(*P*‐value)].

To further explore which regulatory pathways are involved in the LARGE1‐PGL2 module, we performed RNA‐seq analysis on young panicles of ZH11 (wild type, WT), *large1‐1* mutants and *PGL2‐OE#1* overexpression lines. This enabled a comprehensive comparison of differentially expressed genes (DEGs) between the *large1‐1* and *PGL2‐OE#1*. Transcriptome profiling identified a total of 6735 DEGs in *large1‐1* and 9317 DEGs in *PGL2‐OE#1* relative to WT (Figure 5A,B). Strikingly, we observed significant overlap between LARGE1‐regulated and PGL2‐regulated genes. Among the 3800 transcripts upregulated in *large1‐1*, 952 (25%) were also upregulated in *PGL2‐OE#1*, Similarly, 1051 of the 2935 transcripts downregulated in *large1‐1* (36%) were downregulated in *PGL2‐OE#1* (Figure 5A,B). These results indicated that *LARGE1* and *PGL2* share overlapping downstream genes.

Within these overlapping genes, the expression of several Brassinosteroid (BR) signaling‐related genes, including *OsBRL1*, *OsBZR1*, *DLT*, *DLT2*, *OFP3, OsOFP19*, *LO9‐177*, *GL3.1*, and *BU1*, was significantly altered in the young panicles of *large1‐1* and *PGL2‐OE#1* compared to WT plants (Figure 5C). *OsBRL1*, which shares similarity with *OsBRI1* (Nakamura et al., 2006), was upregulated in *large1‐1* and *PGL2‐OE#1* (Figure 5C). Similarly, *BZR1*, a key transcriptional regulator in BR signaling that modulates diverse transcription factors (Ikeda et al., 2013; Kim et al., 2017; Wang et al., 2009), was highly upregulated in both *large1‐1* and *PGL2‐OE#1* (Figure 5C). Conversely, the transcript levels of *DLT* and *DLT2* (also named *D26*), negative regulators of grain size and BR signaling (Lin et al., 2019; Sun et al., 2013; Tong et al., 2012; Zou et al., 2023), were significantly downregulated in *large1‐1* and *PGL2‐OE#1* (Figure 5C). *OFP3* (*OVATE FAMILY PROTEIN 3*) functions as a transcriptional repressor, suppressing BR biosynthesis and signaling to negatively regulate cell elongation, thereby modulating grain size. Notably, OFP3 interacts with both GSK2 and DLT to inhibit BR responses, as previously demonstrated (Xiao et al., 2020). Consistent with its role in growth restriction, the expression of *OFP3* was also downregulated in *large1‐1* and *PGL2‐OE#1* (Figure 5C).

In contrast, positive regulators of grain length and BR signaling, *OsOFP19*, *LO9‐177*, *GL3.1*, and *OsBU1*, were significantly upregulated in *large1‐1* and *PGL2‐OE#1*. OsOFP19 antagonizes DLT in BR signaling, and its overexpression results in shorter, wider, and thicker grains in the ZH11 background (Yang, Ma, et al., 2018). The upregulation of *OsOFP19* in *large1‐1* and *PGL2‐OE#1* (Figure 5C) suggests it may counterbalance *DLT* to primarily influence grain width. Similarly, LO9‐177, which forms a protein complex with PGL2/OsBUL1 and OsBC1 to regulate grain size via cell elongation (Jang et al., 2017), showed pronounced upregulation. *GL3.1*, a BR‐responsive gene enhancing cell elongation and division (Zhang et al., 2012), was also upregulated in *large1‐1* and *PGL2‐OE#1* (Figure 5C), consistent with that overexpression of *GL3.1* has been described to increase grain size in the ZH11 background (Liu et al., 2022). Likewise, *OsBU1*, which acts as a positive regulator of grain length and BR signaling, was significantly upregulated in *large1‐1* and *PGL2‐OE#1*.

BR signaling regulators usually suppress BR biosynthesis genes expression through feedback mechanisms (Bai et al., 2007; Duan et al., 2014; Tong et al., 2009). BZR1 inhibits BR biosynthesis genes expression by directly binding to BR response element (BRRE) (He et al., 2005; Tong et al., 2009). In the study, we observed elevated expression of *OsBZR1* in both *large1‐1* and *PGL2‐OE#1* (Figure 5C), alongside significant changes in the expression of three BR biosynthetic genes. Specifically, *CYP734A2*, which reduces endogenous bioactive brassinolide levels by degrading BR precursors (Qin et al., 2018; Sakamoto et al., 2011; Wu et al., 2015), was markedly upregulated in *large1‐1* and *PGL2‐OE#1* compared to WT (Figure 5C). Conversely, *D11/CYP724B1* and *BRD2*, as the positive regulators of BR biosynthesis and grain size, were downregulated (Figure 5C) (Liu et al., 2016; Wu et al., 2015).

Among the overlapping genes, the expression of *ZFP185* (an A20/AN1‐type zinc finger gene) and *OsGASR1* (a GA‐stimulated transcript‐related gene) was downregulated, both of which negatively regulate cell size (Qin et al., 2018; Sakamoto et al., 2011; Wu et al., 2015). Conversely, some key positive regulators of cell or grain length – including *OsGatB* (a glutamyl‐tRNA amidotransferase B subunit gene), *WTG1* (*wide and thick grain 1*), *OsABC1‐7* (an ABC1‐like kinase gene), *GAD1* (*GRAIN LENGTH AND AWN DEVELOPMENT1*), and *MHZ7/OsEIN2* – were significantly upregulated in the young panicles of *large1‐1* and *PGL2‐OE#1* plants (Figure 5C) (Huang et al., 2017; Jin et al., 2016; Li et al., 2015; Ma et al., 2013; Qin et al., 2016). Grain filling, a critical determinant of grain size and weight, was also affected in these lines. Specifically, the expression of *GIF1* (*GRAIN INCOMPLETE FILLING 1*) and *GIF2* (*GRAIN INCOMPLETE FILLING 2*), two genes involved in regulating grain filling, was downregulated in the young panicles of *large1‐1* and *PGL2‐OE#1* (Tang et al., 2016; Wang, Wang, et al., 2008; Wei et al., 2017). These findings further suggest that *LARGE1* and *PGL2* may work antagonistically within a common pathway to modulate rice grain size.

### APG can bind to the promoter of *OFP3* and promote its transcription

Given that LARGE1 can alleviate the inhibitory effect of PGL2 on APG's transcriptional activation activity, we sought to identify the downstream target genes directly regulated by APG. As a transcription factor, *APG* is predicted to bind promoter elements of its target genes. We therefore investigated whether the promoters of grain size‐related genes, whose expression was significantly altered in the young panicles of *large1‐1* and *PGL2‐OE#1* plants, contained putative APG binding sites. Based on previous studies, APG (also known as OsPIL16) binds to the N‐box motif [CACG(C/A)G] (He et al., 2016).

Our analysis revealed that the promoter regions of *OFP3* contain the N‐box sequences: CACGCG and CACGAG by FIMO scans (Grant et al., 2011). *OFP3* acts as a transcriptional repressor that negatively regulates grain size by suppressing brassinosteroid biosynthesis and signaling, thereby inhibiting cell elongation (Xiao et al., 2020). Consistent with its role as a growth repressor, *OFP3* expression was downregulated in both *large1‐1* and *PGL2‐OE#1* mutants (Figure 5C). We therefore performed electrophoretic mobility shift assay (EMSA) to test whether APG directly binds these N‐box motifs in the *OFP3* promoter. As shown in Figure S8, GST‐APG bound to a biotin‐labeled probe containing the CACGCG sequence, but not to probes with mutated sequences. This demonstrates that APG directly binds the *OFP3* promoter.

To further investigate whether APG regulates the transcription of *OFP3*, we conducted the promoter activity assay in *N. benthamiana*. Through the dual‐luciferase reporter assay, we found that overexpression of APG can significantly enhance the activity of the *OFP3* promoter, resulting in the upregulation of the *LUC* reporter gene (Figure S9). Collectively, APG binds to the *OFP3* promoter and activates *OFP3* transcription. Therefore, the observed downregulation of *OFP3* expression in both the *large1‐1* mutant and *PGL2‐OE#1* overexpression line is consistent with its established role.

## DISCUSSION

Regulation of grain size is a fundamental issue in increasing cereal grain yield. Nevertheless, the molecular and genetic mechanisms governing grain size remain incompletely characterized. We previously reported that the GSK2 protein kinase phosphorylates and stabilizes an RNA‐binding protein OML4/LARGE1 to suppress grain growth (Lyu et al., 2020). However, it remains unclear how *LARGE1* regulates grain size in rice. In this study, we discovered that LARGE1 physically interacts with PGL2 (Figure 1), an atypical HLH transcription factor previously implicated in grain size regulation through promotion of cell expansion in the spikelet hull (Figure S1) (Heang & Sassa, 2012b; Jang et al., 2017). Notably, *LARGE1* restricts grain size primarily by limiting cell expansion in the spikelet hull (Lyu et al., 2020). Genetic analyses revealed that *LARGE1* and *PGL2* act within a common pathway to coordinately regulate both grain length and width in rice (Figure 4). Furthermore, GSK2 is known to regulate LARGE1 stability (Lyu et al., 2020). Consistent with this, our genetic analyses supported that *GSK2* and *PGL2* have overlapped function in grain size control (Figure S11). Transcriptomic profiling (RNA‐seq) further supports their overlapping roles in grain size control, with significant concordance in differentially expressed genes between *large1‐1* and *PGL2‐OE#1* (Figure 5A–C). Our study demonstrates that LARGE1 interacts with PGL2 and competitively represses the interaction of PGL2 and APG, thereby alleviating the inhibition of PGL2 on the transcription activation activity of APG.

In rice, bHLH transcription factors are classified into two major groups (Li et al., 2006): one group includes bHLH proteins containing a DNA‐binding basic region, while the other group consists of HLH proteins lacking this domain, rendering them unable to bind DNA. Atypical HLH proteins, such as those described here, are proposed to function primarily by inhibiting the typical bHLH transcription factors through the formation of non‐functional heterodimers (Massari & Murre, 2000; Sun et al., 1991). Several atypical bHLH proteins, such as BZR1, PGL1, PGL2/OsBUL1, OsIBH1, ILI1/OsbHLH154, BU1/OsbHLH172, OsbHLH079, OsbHLH92, OsbHLH98, OsbHLH107, OsbHLH157, OsbHLH158, OsAIF1/OsbHLH176, and OsAIF2/OsbHLH178, have been implicated in regulating cell elongation and expansion in specific organs, likely via heterodimerization with other typical bHLH partners (Bai et al., 2007; Guo et al., 2021; Heang & Sassa, 2012a, 2012b; Jang et al., 2017; Lu et al., 2025; Tanaka et al., 2009; Teng et al., 2023; Yang, Ren, et al., 2018; Zhang et al., 2009). For example, PGL2 antagonizes the bHLH protein APG to regulate rice grain size by promoting cell expansion (Heang & Sassa, 2012a, 2012b, 2012c). However, a previous study reported no detectable interaction between PGL2 and APG in yeast cells (Jang et al., 2017). In contrast, our findings demonstrate the interaction between PGL2 and APG in Y2H assays (Figure S2). Further validation using firefly luciferase complementation assays and *in vitro* pull‐down experiments confirmed this interaction (Figure 2A). Dual‐luciferase assays further revealed that PGL2 suppresses the transcriptional activation activity of APG by forming a PGL2/APG heterodimer (Figure 3B). Given that APG can homodimerize (Heang & Sassa, 2012a, 2012b), we hypothesized that LARGE1 might disrupt the formation of the PGL2/APG heterodimer by competitively binding PGL2. Consistent with this model, we observed that LARGE1 represses the interaction between PGL2 and APG by competitively binding PGL2 (Figure 2A). This competitive interaction influences the transcriptional activation activity of APG (Figure 3B). Elevated LARGE1 protein levels progressively counteract the inhibitory effect of PGL2 on APG. Collectively, our study highlights an antagonistic regulatory system in which HLH and bHLH proteins modulate rice grain size. This work uncovers a conserved mechanism wherein a pair of antagonistic HLH/bHLH transcription factors, acting downstream of LARGE1, fine‐tune grain development through competitive protein interactions.

Transcription factors (TFs) are well‐established master regulators of gene transcription, while RNA‐binding proteins (RBPs) are traditionally viewed as mediators of post‐transcriptional gene regulation. However, emerging evidence suggests these processes are functionally interconnected, enabling dynamic crosstalk between transcriptional and post‐transcriptional mechanisms mediated by regulatory RNAs and RBPs (Du & Xiao, 2020). RBPs and TFs collaborate through multiple mechanisms – ranging from direct participation in transcription complexes to modulation of mRNA stability and translation efficiency – thereby collectively shaping gene expression networks (Xiao et al., 2019). For example, the DEAD‐box RNA‐binding protein p68 (DDX5) physically interacts with the transcription factor CTCF to regulate chromatin architecture (Yao et al., 2010). Similarly, the RNA‐binding protein RBM25 interacts with the TF YY1 to mediate its transcriptional functions (Xiao et al., 2019). In plants, FCA, an RNA‐binding protein, binds to the mRNA precursor of FLC (a transcription factor that inhibits flowering) and partners with FY, an RNA 3′ end processing factor, to promote premature polyadenylation and degradation of FLC transcripts (Simpson et al., 2003). This alleviates repression of flowering promoters (e.g., *FT*, *SOC1*), accelerating Arabidopsis flowering (Manzano et al., 2009; Simpson et al., 2003). Similarly, PIBP1, an RRM protein, works with a transcription factor Os06g02240 to regulate blast resistance in rice (Zhai et al., 2019).

RNA‐binding proteins (RBPs) are crucial regulators of post‐transcriptional gene expression, involved in processes such as mRNA processing, export, localization, translation, and degradation (Castello et al., 2012; Duarte‐Conde et al., 2022; Gebauer et al., 2021; Gerstberger et al., 2014). Most RBPs contain conserved RNA‐binding domains, including the RNA recognition motif (RRM), KH domain, and others, which confer binding affinity and specificity (Lunde et al., 2007). The RRM, in particular, exhibits high RNA‐binding affinity, and the presence of multiple RRMs can significantly enhance binding strength (Cléry et al., 2008; Maris et al., 2005). *LARGE1* encodes a Mei2‐like protein with three RRM domains. *In vitro* RNA‐binding assay verified that LARGE1 can directly bind to RNA (poly U) with its C‐terminal RRM domain (Figure S10b). It will be a challenge to investigate whether the RNA‐binding activity of LARGE1 contributes to the regulatory pathway described in this study.

**Figure 6:**
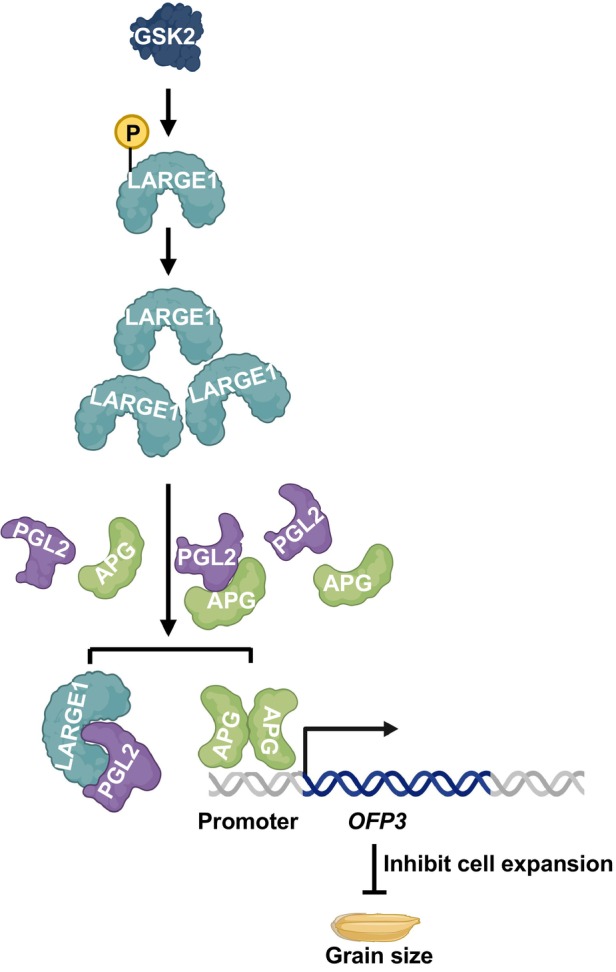
A possible working model for GSK2‐LARGE1‐PGL2/APG module in regulating grain size in rice. Normally, GSK2 phosphorylates LARGE1, thereby increasing LARGE1 protein abundance. Enhanced LARGE1 protein level competes with APG for binding to PGL2, which alleviates the inhibition of PGL2 on the transcriptional activation activity of APG. As a transcriptional activator, APG promotes the expression of *OFP3 –* a negative regulator of grain size in rice – resulting in smaller grains.

Building on these precedents and our molecular and genetic evidence, we propose a possible working model for the GSK2‐LARGE1‐PGL2/APG regulatory module in controlling grain size in rice (Figure 6). In this model, GSK2 phosphorylates the RNA‐binding protein LARGE1, enhancing its protein stability (Lyu et al., 2020). LARGE1 subsequently represses the formation of the PGL2/APG heterodimer by competitively binding to PGL2, thereby promoting APG homodimer accumulation and enhancing its transcriptional activation activity (Figure 3B). RNA‐seq analysis of young panicles of *PGL2‐OE* and *large1‐1* mutants revealed altered expression of multiple genes associated with BR biosynthesis and signaling (Figure 5). Consistent with this, *large1* and *pgl2* mutants exhibit BR‐related phenotypic defects (Lyu et al., 2020). Collectively, our study reveals a previously unrecognized mechanism by which the GSK2‐LARGE1‐PGL2/APG regulatory module controls grain size, and underscores the broader significance of RBP‐TF interplay in transcriptional control (Figure 6). This pathway represents a promising target for improving seed size and weight in crops.

## MATERIALS AND METHODS

### Plant materials and growth conditions

The rice varieties selected for this study comprised ZH11 (*Oryza sativa* L. ssp. *Japonica* cv. ZhongHua11), along with derived mutants and transgenic lines: *large1‐1* (obtained through gamma‐ray mutagenesis of ZH11 M_2_ progenies), *pgl2‐cri* (CRISPR‐Cas9‐generated PGL2 mutants in ZH11), *PGL2‐OE* (ZH11 transformants with *PGL2* overexpression), *GSK2‐RNAi* (ZH11 lines exhibiting *GSK2* suppression via RNAi), 35S:MYC‐PGL2 (constitutive PGL2‐overexpressors with MYC‐tag), and *35S:GFP‐LARGE1* (*LARGE1*‐overexpressors with GFP fusion). All plant materials were grown in standard paddy field conditions using a 20 × 20 cm planting grid. Fertilization management included balanced application of nitrogen‐based (N), phosphorus‐containing (P), and potassium‐supplemented (K) compounds, each administered at 120 kg ha^−1^ across developmental stages. The *large1‐1* mutant was isolated from gamma rays‐irradiated ZH11 M_2_ population (Lyu et al., 2020). Wild type ZH11, along with mutant lines *large1‐1*, *pgl2‐cri*, *PGL2‐OE*, and other rice varieties, were cultivated under field conditions following established protocols (Huang et al., 2017). The experimental trials were conducted from December to April in subsequent growing seasons at the designated research station located in Lingshui County, Hainan Province, China. From May to October, these rice plants were cultivated in the experimental plots at Changping District, Beijing, China.

*N. benthamiana* plants were grown in potting medium within a controlled environment chamber set to maintain constant temperature (22 ± 2°C). During the cultivation phase spanning approximately 5 weeks, plants received daily photosynthetic photon flux density (PPFD) of 120 μmol m^*−*2^ sec^*−*1^ under a 16‐h light/8‐h dark cycle regimen. For transient expression assays, mature leaf pairs corresponding to developmental positions three and four (completely expanded) were selected for experimental procedures.

### Morphological evaluation

Take photographs of the plants and panicles after the rice has fully matured. Mature seeds harvested from primary panicles were subjected to digital imaging using a scanner (Scan Marker i560; MICROTEK International Inc., Shanghai, China, http://www.microtek.com/). Subsequently, seed morphological parameters, including longitudinal and transverse dimensions, were automatically quantified through computational analysis via the WSeen Rice Evaluation Platform (Hangzhou WSeen Technology Co., Ltd., Hangzhou, Zhejiang, China, http://www.wseen.com/). The 1000‐kernel weight of dehydrated seeds was quantified through multiple weighing trials (≥3) using calibrated laboratory balances. The primary panicles of individual plants were utilized to quantify primary and secondary branch counts, as well as grain numbers per panicle.

### Real‐time PCR analysis

Total RNA was extracted from 7‐day‐old seedlings of wild type ZH11 and *PGL2‐OE* transgenic lines employing the RNAprep Pure Plant Kit (Tiangen, Beijing, China, Cat# DP432). The complementary DNA was generated in accordance with the guidelines provided by the manufacturer for the ThermoScript™ reverse transcription‐PCR kit (Therom Fisher Scientific, MA, USA；EP0441). Subsequent quantitative PCR amplifications were conducted with SYBR® Premix Dimer Eraser™ chemistry (Takara Bio, Kusatsu, Shiga, Japan；Cat# DRR091A), employing the Applied Biosystems 7500 detection system (Thermo Fisher Scientific, Waltham, MA, USA) for real‐time fluorescence monitoring. For data standardization across experimental samples, *OsActin1* expression levels were measured as an internal control gene. The complete set of oligonucleotide primers used in these quantitative reverse transcription‐PCR experiments has been detailed in Table S1.

### Plasmid construction and plant transformation

The coding sequence of *PGL2* was amplified through PCR amplification employing primers PGL2‐OE‐F and PGL2‐OE‐R with cDNA obtained from young panicle tissues of the ZH11 cultivar as template. The amplified fragment was subsequently subcloned into the pIPKB003 binary vector to generate the *proActin:PGL2* recombinant plasmid, where the transgene expression is regulated by the constitutive *Actin* promoter. For the *35S:MYC‐PGL2* construct, full‐length cDNA containing the *PGL2* open reading frame was synthesized through PCR amplification with MYC‐PGL2‐F/MYC‐PGL2‐R primers using the same plant material, followed by directional insertion into the pCAMBIA1300‐221‐MYC vector via seamless recombination. The *35S:GFP‐LARGE1* construct was generated through PCR‐mediated amplification of the *LARGE1* coding sequence (CDS) using GFP‐LARGE1‐F/R primers, with cDNA obtained from juvenile panicle tissues of ZH11 as the template. The amplified product was subsequently cloned into the pMDC43 vector via seamless recombination. All molecular cloning procedures were performed with the ClonExpress II One Step Cloning System (Vazyme Biotech Co., Ltd, Nanjing, China; Cat# C112‐01). For genome modification, two single‐guide RNA (sgRNA) constructs were developed to hybridize with 5′ initiation segments of distinct *PGL2* exons, followed by commercial synthesis through BGI Tech Solutions (Shenzhen, China). Each synthesized sgRNA cassette was separately cloned into the SK‐gRNA transfer vector via T_4_ DNA ligase (New England Biolabs, Ipswich, MA, USA；Cat#0202S). Through restriction‐ligation cloning methodology, these modular vectors were subsequently combined with the pC1300‐Cas9 plant expression framework to assemble the complete binary vector *pC1300‐Cas9‐PGL2* (Shan et al., 2013).

The engineered constructs above were, respectively, electroporated into chemically competent *Agrobacterium tumefaciens* GV3101 cells. Following established *Agrobacterium*‐mediated transformation procedures (Hiei et al., 1994), the recombinant bacterial suspensions were inoculated onto embryogenic calli derived from *Oryza sativa* cv. ZH11 to establish transgenic plant lines, including *PGL2‐OE*, *35S:MYC‐PGL2*, *35S:GFP‐LARGE1*, and *pgl2‐cri* mutants. sgRNA targeting sequences are illustrated in Figure 2(A), with corresponding primer sequences documented in Table S1.

### Yeast two‐hybrid assay

PCR amplification was conducted on the coding sequences of *LARGE1* and *PGL2* genes using cDNA obtained from immature panicles of the japonica rice cultivar ZH11 as template. These amplified products were subsequently cloned into pPR3N and pDHB1 destination vectors through In‐Fusion Cloning Kit (Vazyme Biotech Co., Ltd, Nanjing, China; Cat# C112‐01), respectively. As outlined in the DUAL MEMBRANE KIT manufacturer's guidelines (Dualsystems Biotech, Schlieren, Ssa, witzerland; Cat. No. K20303‐1), both recombinant plasmids were simultaneously introduced into *Saccharomyces cerevisiae* strain NMY51 via lithium acetate transformation. The transformed yeast was grown on SD agar plates lacking leucine and tryptophan for selection, with subsequent incubation at 30°C over a 72‐h period. For interaction validation, colonies grown on primary selection plates were resuspended in sterile saline (0.9% NaCl) and subsequently replica‐plated onto secondary screening media (SD/−Leu−Trp−His−Ade; Takara Bio, Kusatsu, Shiga, Japan; Cat#  630317) to assess protein–protein interaction through auxotrophic marker complementation.

### Luciferase complementation imaging assay

The *PGL2* coding sequence was PCR‐amplified with specific primers PGL2‐nLUC‐F/PGL2‐nLUC‐R and introduced into the pCAMBIA1300‐35S‐nLUC‐RBS vector backbone (Chen et al., 2008) through seamless cloning, employing the ClonExpress II system (Vazyme Biotech Co., Ltd, Nanjing, China; Cat# C112‐01) for seamless assembly to create the *PGL2‐nLUC* construct. Following a similar strategy, *LARGE1* and *APG* genes were cloned using distinct primer combinations: the *LARGE1* coding sequence was amplified with the cLUC‐LARGE1‐F/R primer set and directionally cloned into the pCAMBIA1300‐35S‐cLUC‐RBS plasmid, whereas *APG* was amplified using the cLUC‐APG‐F/R primer pair and subsequently inserted into identical vector backbones, ultimately producing the *cLUC‐LARGE1* and *cLUC‐APG* recombinant plasmids, respectively.

The constructed plasmid vectors were electroporated into *Agrobacterium tumefaciens* strain GV3101 for bacterial transformation. Transient expression assays in plants were conducted according to published methodology (Chen et al., 2008) with specific adjustments: Transfected *Agrobacterium* cultures were first incubated at 28°C for 12–16 h, after which equivalent amounts of individual bacterial suspensions (standardized to OD_600_ = 0.6 in an induction solution containing 10 mm MgCl_2_, 150 μm acetosyringone, and 10 mm MES adjusted to pH 5.6) were combined. The mixed cultures were then subjected to continuous low‐speed agitation at 25°C for 2–5 h to enhance virulence induction. For plant tissue inoculation, the prepared bacterial solutions were introduced into fully expanded leaves of *Nicotiana benthamiana* plants through syringe‐mediated vacuum infiltration. Luciferase activity measurements were performed following standard analytical procedures.

Post‐infiltration plants were maintained at 22°C for 48 h under controlled conditions. For luminescence detection, excised leaves were uniformly coated with 1 mm d‐luciferin (Promega, Madison, WI, USA; Cat# E1602) solution on the abaxial surface and dark‐adapted for 2–5 min at ambient temperature. Luminescent signals were captured using a NightOWL II LB983 imaging system (Berthold Technologies, Bad Wildbad, Germany) equipped with Indigo software. Quantitative measurements for competition assays employed both CCD imaging and microplate luminometry (GloMax® 96 Microplate Luminometer, Promega, USA; Cat# E6501) with 96‐well plate formats. All primer sequences are provided in Table S1.

### Bimolecular fluorescence complementation assay

The complete coding region of *PGL2* was fused to the N‐terminal domain of YFP through a PCR‐based assembly approach employing primer pairs PGL2‐nYFP‐F1/R1 and PGL2‐nYFP‐F2/R2. These amplified products were subsequently inserted into the binary plant expression vector pGBW414, creating the recombinant plasmid designated as *pGBW‐PGL2‐nYFP*. In parallel, the *LARGE1* coding sequence was directionally linked to the C‐terminal portion of YFP (utilizing amplification primers LARGE1‐cYFP‐F1/R1 and LARGE1‐cYFP‐F2/R2) followed by subcloning into the same vector system to produce the *pGBW414‐LARGE1‐cYFP* construct. All primer sequences are detailed in Table S1.

These engineered plasmids were subsequently introduced into *Nicotiana benthamiana* leaf epidermal cells via *Agrobacterium*‐mediated transformation, employing the transient expression protocol described previously. The protein interaction activity was monitored through laser scanning confocal microscopy at a 48‐h interval after infiltration. Fluorescent emissions were measured under 488 nm excitation parameters, with high‐resolution imaging data systematically collected for comprehensive evaluation.

### *In vitro* pull‐down assays

The coding sequence of *LARGE1*, *PGL2*, and *APG* were amplified and inserted into pETnT, pGEX4T1, and pMALC2 vectors for the purification of FLAG‐LARGE1, FLAG‐APG, GST‐PGL2, and MBP‐LARGE1 recombinant proteins (amplifying primers were FLAG‐LARGE1‐F and FLAG‐LARGE1‐R, FLAG‐APG‐F and FLAG‐APG‐R, GST‐PGL2‐F and GST‐PGL2‐R, MBP‐LARGE1‐F and MBP‐LARGE1‐R) (Peleg & Unger, 2012). To examine the interaction between LARGE1 and PGL2, equal amounts of FLAG‐LARGE1 and GST‐PGL2/GST were incubated in TGH buffer (50 mm HEPES, pH 7.5, 10% glycerol, 150 mm NaCl, 1% Triton X‐100, 1.5 mm MgCl_2_, 1 mm EGTA, and 1 tablet of protease inhibitor cocktail for 40 ml TGH buffer) and mixed with glutathione agarose (Thermo Fisher Scientific, Waltham, MA, USA; Cat# 16100) at 4°C with gentle shaking for 0.5–2 h. Following low‐speed centrifugation (25 ***g***, 2 min), the resin pellets were harvested and subsequently subjected to three washing cycles. The washing buffer was prepared by combining 50 mm HEPES buffer (pH 7.5) with 150 mm NaCl, 1.5 mm MgCl_2_, 1% Triton X‐100, 10% glycerol, and 1 mm ethylene glycol tetraacetic acid (pH adjusted to 8.0). Immediately before application, one protease inhibitor cocktail tablet was dissolved per 40 ml of the prepared solution. For protein analysis, bead‐bound complexes were mixed with SDS‐loading buffer (50 mm Tris–HCl pH 6.8, 2% SDS, 10% glycerol, 0.1% bromophenol blue) containing 1% β‐mercaptoethanol, followed by thermal denaturation at 95°C for 5 min. Protein separation was performed using 10% SDS‐PAGE, after which electrophoretic transfer onto PVDF membranes (Merk Millipore, Billerica, MA, USA; Cat# ISEQ00010) was conducted for immunodetection. Sequential membrane incubations were carried out using: (1) FLAG‐specific antibodies (Abmart, Shanghai, China; Cat# M20008, diluted 1:5000) and (2) GST‐targeting antibodies (Abmart, Shanghai, China; Cat# M20007, diluted 1:5000; Abma). All immunoblotting steps followed standard protocols for glutathione‐immobilized protein analysis.

To assess competitive binding between LARGE1 and APG, equal quantities of FLAG‐APG and GST‐PGL2 fusion proteins were combined with incremental concentrations of MBP‐LARGE1 (1×, 2×, and 5× equivalents) prior to glutathione resin incubation. FLAG‐APG retained on the beads was subsequently analyzed by anti‐FLAG immunoblotting. Signal detection was performed using the eECL Western Blot Kit (Cwbiotech, Beijing, China; Cat# CW0049), followed by digital imaging with a Tanon 4500 automated gel documentation system (Tanon Science & Technology, Shanghai, China).

### *In vivo* co‐immunoprecipitation

To obtain dual transgenic lines of *35S:MYC‐PGL2;35S:GFP* and *35S:MYC‐PGL2;35S:GFP‐LARGE1*, established *35S:MYC‐PGL2* plants served as the maternal parent in crosses with verified *35S:GFP* and *35S:GFP‐LARGE1* transgenic lines. To conduct protein extraction and characterization, both shoot and root tissues harvested from 5‐day‐old hybrid seedlings were mechanically disrupted in an ice‐cold lysis solution composed of: 150 mm NaCl, 50 mm Tris–HCl (pH‐adjusted to 7.5), 2% (v/v) Triton X‐100 detergent, 20% (v/v) glycerol, 1 mm EDTA chelator, 1 mm phenylmethylsulfonyl fluoride (PMSF), along with a commercially available protease inhibition cocktail. Cellular lysates were subjected to affinity purification using GFP‐Trap^®^ Agarose beads (ChromoTek, Planegg‐Martinsried, Germany, Cat# GTA‐20) through 40‐min rotational incubation at 4°C. The beads underwent four sequential washing cycles using a modified buffer solution where Triton X‐100 concentration was progressively reduced from 2% to 0.1%, while maintaining other components identical to those in the lysis buffer. Immunoblot detection employed anti‐MYC monoclonal antibodies (Abmart, Shanghai, China; Cat# M20002m) to verify MYC‐PGL2 presence in eluted fractions.

### Rice protoplast preparation and transcriptional activity assay

Rice protoplast isolation and transformation procedures were conducted using an adapted protocol based on the approach outlined previously (Zhang et al., 2011). Mature ZH11 seedlings were dark‐cultivated for 10–14 days prior to tissue harvesting. The stem‐sheath junction regions were dissected into approximately 0.5 mm longitudinal sections. The collected tissue samples were immediately placed into a digestion solution consisting of 1.5% weight/volume cellulose RS, 0.75% macerozyme R‐10, and 0.6 m mannitol, with the mixture further supplemented by 10 mm MES buffer (pH adjusted to 5.7), 10 mm calcium chloride, and 0.1% BSA. The digestion process included 30‐min vacuum infiltration followed by 4‐h incubation and enzymatic hydrolysis under dark conditions with gentle shaking. The prepared mixture was combined with an equal volume of W5 solution (containing 154 mm sodium chloride, 5 mm potassium chloride, 125 mm calcium chloride, and 2 mm MES buffer maintained at pH 5.7). The combined solution underwent intense horizontal agitation lasting 10 sec after blending, aiming to enhance the release of protoplast cells. Then the protoplasts were collected in 50‐ml round‐bottomed centrifuge tubes by filtering the mixture through 22–25 μm Miracloth membranes and centrifugation at 150 ***g*** for 5 min (25°C). Following centrifugation, harvested protoplasts were subjected to two rounds of W5 buffer rinsing prior to resuspension in MMG medium containing 0.4 m mannitol, 15 mm magnesium chloride, and 4 mm MES (pH adjusted to 5.7), attaining a final cellular density of 2.5 × 10^6^ cells ml^−1^. The transformation procedure involved combining 100 μl of prepared protoplast suspension with 5 μg of plasmid vector, followed by addition of 110 μl polyethylene glycol‐based transformation cocktail (40% PEG4000, 0.4 m mannitol, 100 mm calcium chloride). Following 15‐min dark incubation at 28°C, the reaction was stopped with gentle adding 1.8 ml W5 buffer. After centrifugation, transformed protoplasts were maintained in 1 ml W5 buffer under dark conditions at 28°C for 16–18 h prior to subsequent analyses.

The transcriptional activity assay was performed following established methodology (Hao et al., 2011). The pLUC reporter plasmid was engineered to harbor a truncated 35S promoter element (encompassing the TATA box) coupled with five consecutive GAL4 binding motifs (5 × GAL4), initiating expression of the firefly *luciferase* (*LUC*) transgene. In parallel control configurations, the pGAL4 backbone containing the GAL4 DNA‐binding domain (GAL4DBD) coding sequence driven by the constitutive *35S promoter* served as the baseline effector plasmid. To establish functional test constructs, the *APG* coding sequence was cloned into the pGAL4 vector creating pAPG. For comparative activation assessment, the potent transcriptional activator VP16 was combined with GAL4DBD to produce positive control plasmid pVP16. Distinct effector variants *pPGL2* and *pLARGE1* were generated through substitution of GAL4DBD in *pGAL4* with *PGL2* and *LARGE1* coding sequences, respectively. Normalization was achieved using reference plasmid pTRL, which expresses Renilla luciferase under regulatory control of the Arabidopsis *Ubiquitin3* promoter. During rice protoplast transformation experiments, this calibration plasmid was co‐transformed with experimental constructs at a molar ratio of 1:6 (control:test). The transformation was performed with at least 12 biological replicates for each analysis to ensure statistical validity. The luciferase activity was assessed by employing the Dual‐Luciferase^®^ Reporter Assay System (Promega, Madison, WI, USA; Cat# E1500), with luminescence detected through a dual‐injector GloMax^®^96 microplate luminometer.

### RNA‐seq and bioinformatical analysis

Eighteen to twenty‐four young panicles (5 ± 0.1 cm) of wild type ZH11, *large1‐1*, and *PGL2‐OE#1* were harvested and pooled into three samples (≥6 panicles per sample), respectively. Total RNA extraction from all samples was carried out utilizing the RNAprep Pure Plant Kit (Tiangen Biotech, Beijing, China; Cat# DP432) in accordance with the supplier's standardized procedures. RNA integrity evaluation employed two complementary analytical methods: ultraviolet absorbance profiling through a NanoDrop 2000 spectrophotometric system and nucleic acid separation assessment using an Agilent 2100 Bioanalyzer equipped with RNA 6000 Nano analytical chips (Agilent Technologies, Santa Clara, CA, USA; Cat# 5067‐1511). About 2 μg of total RNA was used to create RNA‐seq libraries and then the completed libraries were used for NGS on the Illumina NovaSeq 6000 platform. The trimmed sequencing data were mapped against the IRGSP‐1.0 reference genome (*Oryza sativa japonica* cultivar Nipponbare). Transcript abundance was subsequently calculated and standardized employing TPM normalization. For detection of statistically significant DEGs, we implemented the edgeR software tool (version 3.24) with established significance criteria requiring a minimum twofold or greater differential expression (absolute log_2_‐transformed fold‐change ≥1) combined with BH‐adjusted *P*‐values below 0.05, following previously described statistical methodologies (Robinson et al., 2010). Functional annotation enrichment analyses were performed through dual approaches: KEGG pathway analysis was executed using OmicShare cloud platform (https://www.omicshare.com/tools/), while Gene Ontology (GO) term enrichment for SE‐associated genes was analyzed through TBtools software (v1.120) (Chen et al., 2020). Primer sequences employed for RT‐qPCR validation of SEs have been documented in Table S1.

### Electrophoretic Mobility Shift Assay (EMSA)

EMSA was performed using a commercial kit (LightShift Chemiluminescent EMSA Kit; Thermo Fisher Scientific, Waltham, MA, USA; Cat# 20148). Briefly, GST and GST‐APG fusion proteins were expressed in *Escherichia coli* BL21 (DE3) cells and purified using glutathione agarose (Thermo Fisher Scientific, Waltham, MA, USA; Cat# 16100). Purified proteins were incubated with biotin‐labeled DNA probes (Sangon Biotech, Shanghai, China). For competition assays, unlabeled competitor probes were included in the reaction mixtures. All binding reactions and subsequent detection steps were carried out according to the manufacturer's instructions.

### Promoter activity assay

Promoter activity was assessed using a dual‐luciferase reporter assay system, as previously described (Hellens et al., 2005). A 2000‐bp fragment of the *OFP3* promoter was cloned into the *pGreenII0800‐LUC* vector to generate the *ProOFP3:LUC* reporter construct. The construct was then introduced into *Agrobacterium tumefaciens* strain GV3101 and co‐infiltrated with other specified vectors into leaves of *Nicotiana benthamiana* plants. After incubation at 28°C for 48 h, luminescence was measured using a Luminoskan Ascent Microplate Luminometer (Thermo Fisher Scientific, Waltham, MA, USA). Firefly luciferase (LUC) and Renilla luciferase (REN) activities were quantified with the Dual‐Luciferase® Assay Kit (Promega, Madison, WI, USA; Cat# E1500).

### *In vitro* RNA binding assay

The recombinant proteins MBP‐LARGE1, MBP‐nLARGE1, MBP‐cLARGE1, MBP‐*large1*, and MBP were incubated in RNA‐binding buffer (50 mm Tris–HCl, pH 7.4, 50 mm NaCl, 0.5% Nonidet P‐40) with 40 μl poly(U) beads (Sigma‐Aldrich, St. Louis, MO, USA; Cat# P8563) at 4°C for 1 h. The poly(U) beads were collected after centrifugation at 50 ***g*** for 2 min and washed eight times with ice‐cold washing buffer (same as binding buffer) with gently shaking for 5 min per time. Then the poly(U) beads were resuspended in 50 μl SDS‐loading buffer and denatured at 98°C for 10 min. The recombinant proteins were detected by western blot analysis with the anti‐MBP antibodies (New England Biolabs, Ipswich, MA, USA; Cat# E8032S).

## ACCESSION NUMBERS

Sequence data used in this article can be found in the MSU Rice Genome Annotation Project database under the following accession numbers: *LARGE1* (*LOC_Os02g31290*), *PGL2* (*LOC_Os02g51320*), *APG* (*LOC_Os05g04740*), and *OFP3* (*LOC_Os01g53160*).

## AUTHOR CONTRIBUTIONS

YLi and LZ conceived and designed this project. YaL, LZ, and YLi designed experiments. YaL, LZ, HZ, YG, XX, JL and YZ performed most of the experiments. YaL, LZ, and YLi analyzed data, prepared figures, and wrote the article.

## CONFLICT OF INTEREST

The authors have no competing interests.

## Supporting information


**Figure S1.** Role of *PGL2* in modulating grain size and plant morphological traits in rice.
**Figure S2.** APG exhibits interaction with PGL2.
**Figure S3.** Overexpression of PGL2 affects grain size and multiple morphological traits in rice.
**Figure S4.** Pull‐down assays confirmed the interaction between LARGE1 and PGL2, but not with APG.
**Figure S5.** The interaction between LARGE1 and PGL2 does not affect the subcellular localization of PGL2 or APG.
**Figure S6.** LARGE1 competitively inhibits APG‐PGL2 binding.
**Figure S7.** APG transcriptioanal activation activity is affected by PGL2 and LARGE1.
**Figure S8.** EMSA assay of binding between APG and the predicted potential target gene *OsOFP3*.
**Figure S9.** Dual‐luciferase reporter gene assay validates that APG can enhance the promoter activity of OFP3.
**Figure S10.** RNA‐binding activity of LARGE1 *in vitro* assay.
**Figure S11.** *PGL2* acts genetically with *GSK2*.
**Table S1.** Primers used in this study.

## Data Availability

The raw RNA‐seq datasets generated during the current study are available in the NCBI Sequence Read Archive (SRA) repository, under BioProject accession number PRJNA1391003. The data will be publicly accessible upon publication.
