# Supplementary material for: The LARGE1 controls grain size by repressing the interaction between PGL2 and APG in rice
Source: Plant J. 2026 Jan 16;125(2):e70674. doi: 10.1111/tpj.70674 (PMC12811032; doi:10.1111/tpj.70674)
Supplement: Supplementary file 1 — Figure S1. Role of PGL2 in modulating grain size and plant morphological traits in rice. Figure S2. APG exhibits interaction with PGL2. Figure S3. Overexpression of PGL2 affects grain size and multiple morphological traits in rice. Figure S4. Pull‐down assays confirmed the interaction between LARGE1 and PGL2, but not with APG. Figure S5. The interaction between LARGE1 and PGL2 does not affect the subcellular localization of PGL2 or APG. Figure S6. LARGE1 competitively inhibits APG‐PGL2 binding. Figure S7. APG transcriptioanal activation activity is affected by PGL2 and LARGE1. Figure S8. EMSA assay of binding between APG and the predicted potential target gene OsOFP3. Figure S9. Dual‐luciferase reporter gene assay validates that APG can enhance the promoter activity of OFP3. Figure S10. RNA‐binding activity of LARGE1 in vitro assay. Figure S11. PGL2 acts genetically with GSK2. Table S1. Primers used in this study. [file TPJ-125-0-s001.pdf]

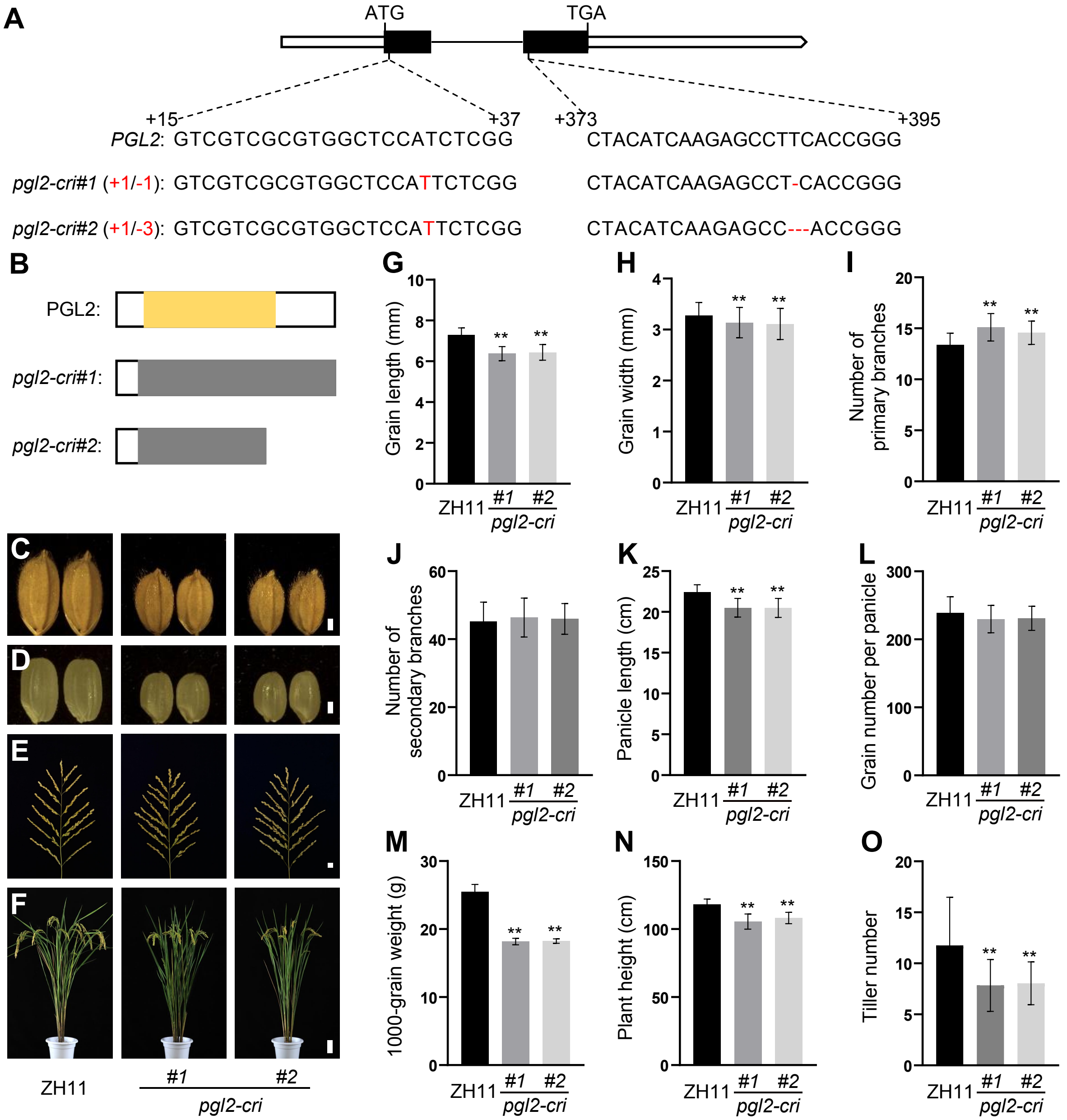

Supplementary Figure 1. Role of *PGL2* in modulating grain size and plant morphological traits in rice

(A) Schematic representation of CRISPR editing sites within the *PGL2* gene structure and modified *pgl2-cri* variants. Vertical black lines positioned above the gene model mark the initiation (ATG) and termination (TGA) codons. Exonic sequences appear as solid rectangles connected by a horizontal line representing the intervening intronic sequence. CRISPR target positions are denoted by the short vertical black lines below the gene structure schematic. Terminal open rectangles identify non-coding regions. Red numerical superscripts (+/-) quantify base insertions and deletions relative to the ZH11 reference. Inserted nucleotides appear in red typeface, while deletion junctions are marked by red dash symbols.

(B) Schematic representation of protein structures of PGL2 and its mutant *pgl2-cri*. The yellow rectangle highlights the HLH domain. The gray rectangle represents the frameshift mutation region in the *pgl2-cri* mutant resulting from gene editing.

(C-D) Morphological comparison of seeds between wild-type ZH11 and *pgl2-cri* mutant lines. Representative images display glume-enclosed grains (C) and dehusked caryopses (D) from both genotypes.

(E) Comparative morphology of rice panicles in ZH11 versus *pgl2-cri* mutant lines.

(F) Comparative morphology of fully developed ZH11 wild-type plants and *pgl2-cri* gene-edited mutant variants.

(G-O) Comparative phenotypic analyses of agronomic traits between wild-type ZH11 and *pgl2-cri* mutant lines, including: grain size (length in G, width in H), panicle architecture parameters (primary branch number in I, secondary branch number in J, main panicle length in K), yield-related characteristics (grains per panicle in L, thousand-seed mass in M), and plant growth features (vertical height in N, tiller number in O).

Data in panels G-O represent mean values with standard deviations (sample size ≥ 25 per group). Significant variations between ZH11 (wild-type) and *pgl2-cri* mutant lines are marked by asterisks: ** denotes $P < 0.01$ and * indicates $0.01 < P < 0.05$ using Student's *t*-test analysis. Scale bars correspond to 1 mm (C-D), 1 cm (E), and 10 cm (F) respectively.

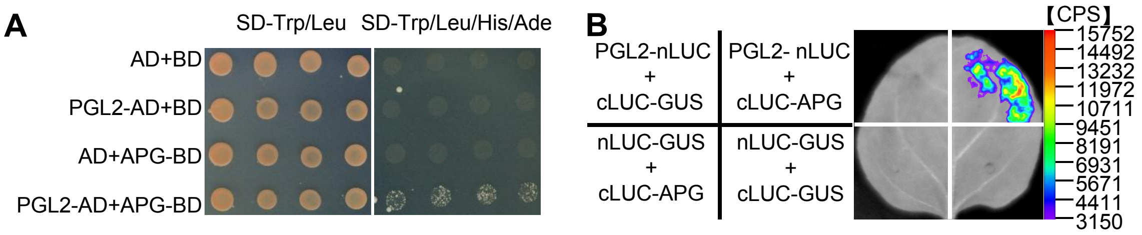

Supplementary Figure 2. APG exhibits interaction with PGL2

(A) Yeast two-hybrid analysis demonstrating APG-PGL2 association. Yeast strains expressing both proteins were grown on selective media (SD/-Trp-Leu) and interaction-confirming media (SD/-Trp-Leu-His-Ade) for viability assessment.

(B) Split-luciferase complementation assay in *N. benthamiana* confirming APG-PGL2 interaction. Leaf tissues co-expressing PGL2-nLUC and cLUC-APG constructs showed reconstituted luciferase activity, visualized through bioluminescence imaging with intensity values represented on a false-color scale.

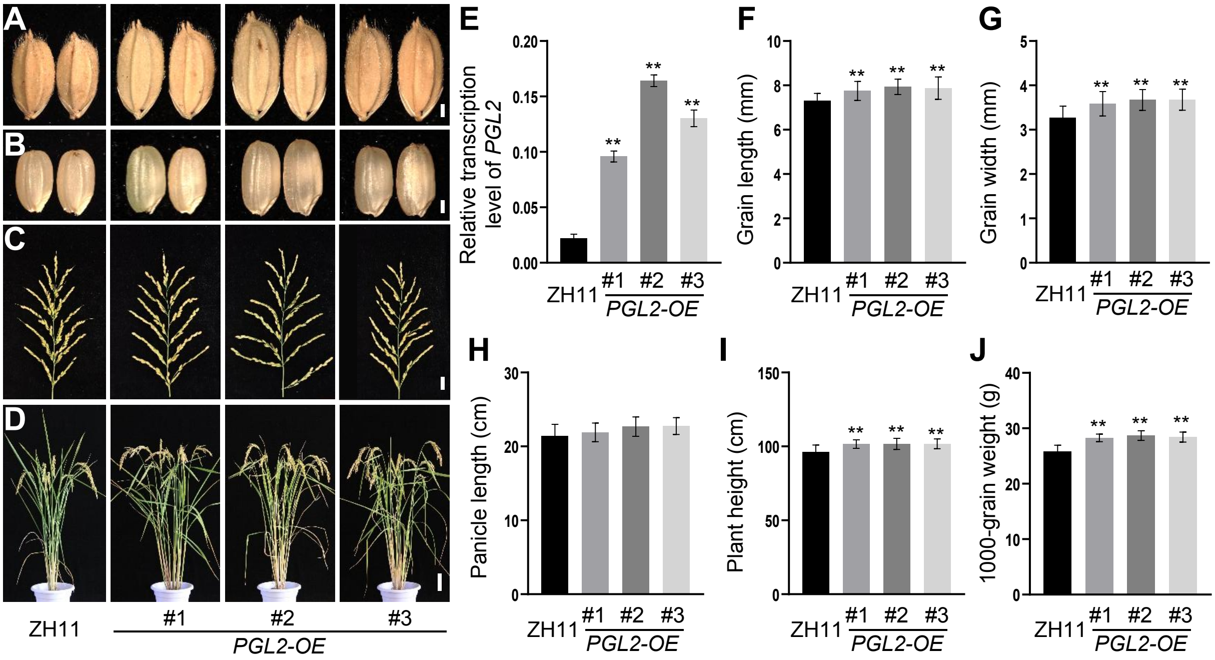

Supplementary Figure 3. Overexpression of *PGL2* affects grain size and multiple morphological traits in rice

(A, B) Grain morphology of ZH11 (wild type) and PGL2-overexpressing (*PGL2-OE*) transgenic lines.

(C) Panicle phenotypes of ZH11 and *PGL2-OE* plants.

(D) Overall plant architecture of ZH11 and *PGL2-OE* lines.

(E) Transcriptional expression levels of *PGL2* in ZH11 and *PGL2-OE* lines.

(F, G) Grain size analysis of ZH11 versus *PGL2-OE* lines.

(H-J) Measurement of panicle length (H), plant height (I), and 1000-grain weight (J) in the two genetic backgrounds.

Data are presented as means ± SD, with sample sizes of n = 4 for (E, N) and n ≥ 30 for (F-M). Significant differences between ZH11 and *PGL2-OE* lines were determined using Student's *t*-test: *$P < 0.05$, **$P < 0.01$ compared to wild type. Scale bars: 1 mm (A, B); 2 cm (C); 10 cm (D).

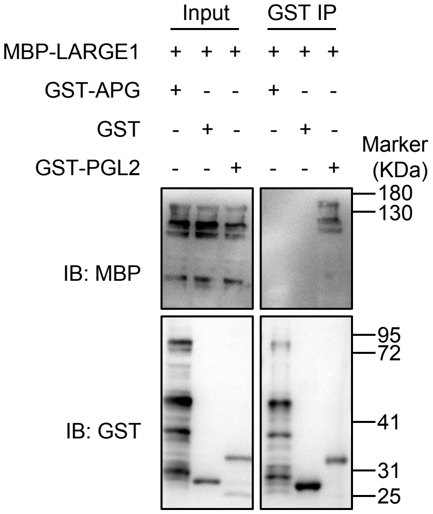

Supplementary Figure 4. Pull-down assays confirmed the interaction between LARGE1 and PGL2, but not with APG

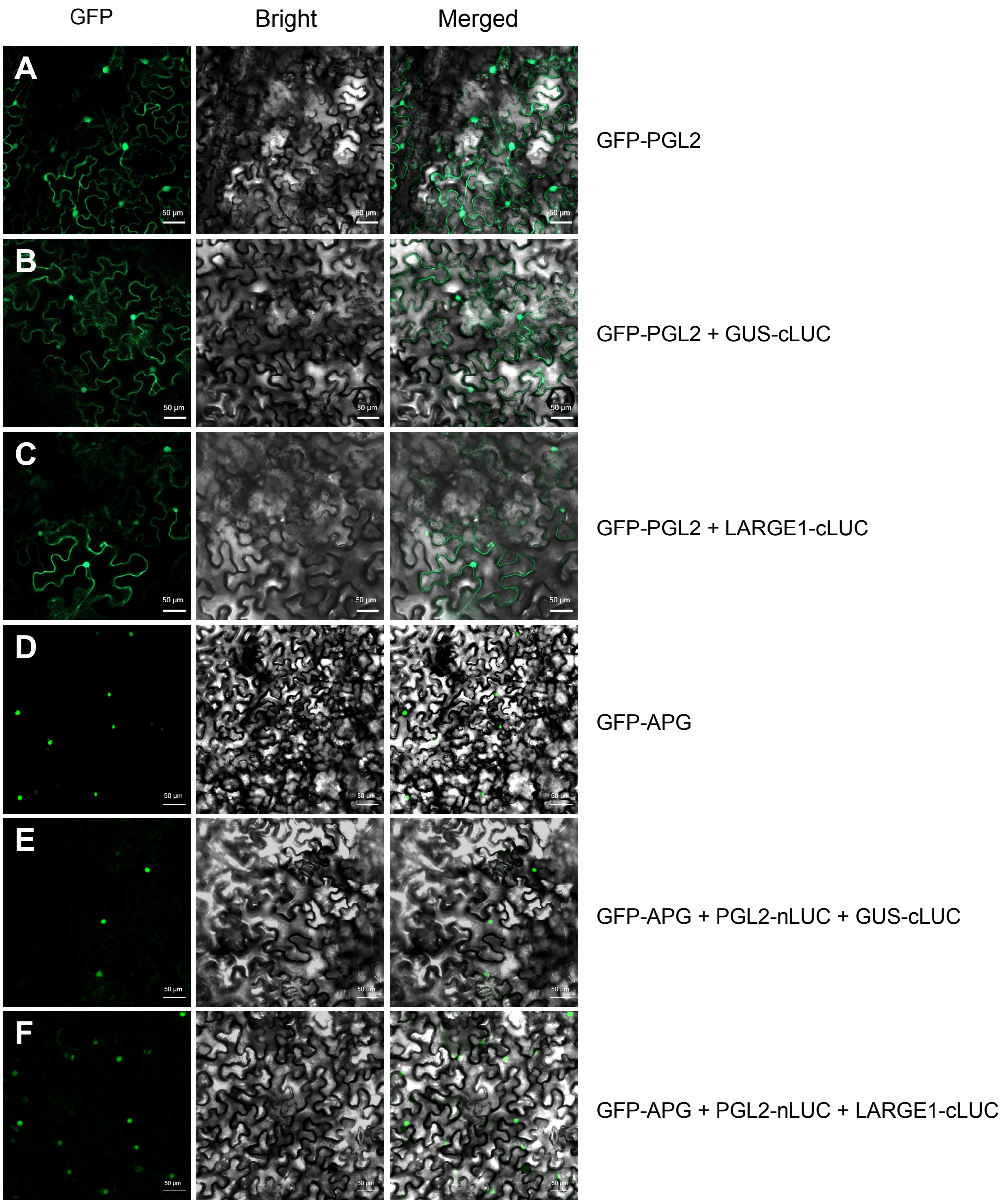

Supplementary Figure 5. The interaction between LARGE1 and PGL2 does not affect the subcellular localization of PGL2 or APG

(A) The subcellular localization of GFP-PGL2 when it co-expressed with LARGE1-cLUC in leaf epidermal cells of *Nicotiana benthamiana*.

(B) The subcellular localization of GFP-PGL2 when it co-expressed with GUS-cLUC in leaf epidermal cells of *Nicotiana benthamiana*.

(C) The subcellular localization of GFP-PGL2 when it expressed singly in leaf epidermal cells of *Nicotiana benthamiana*.

(D) The subcellular localization of GFP-APG when it co-expressed with PGL2-nLUC and LARGE1-cLUC in leaf epidermal cells of *Nicotiana benthamiana*.

(E) The subcellular localization of GFP-APG when it co-expressed with PGL2-nLUC and GUS-cLUC in leaf epidermal cells of *Nicotiana benthamiana*.

(F) The subcellular localization of GFP-APG when it expressed singly in leaf epidermal cells of *Nicotiana benthamiana*.

Scale bars correspond to 50 μm.

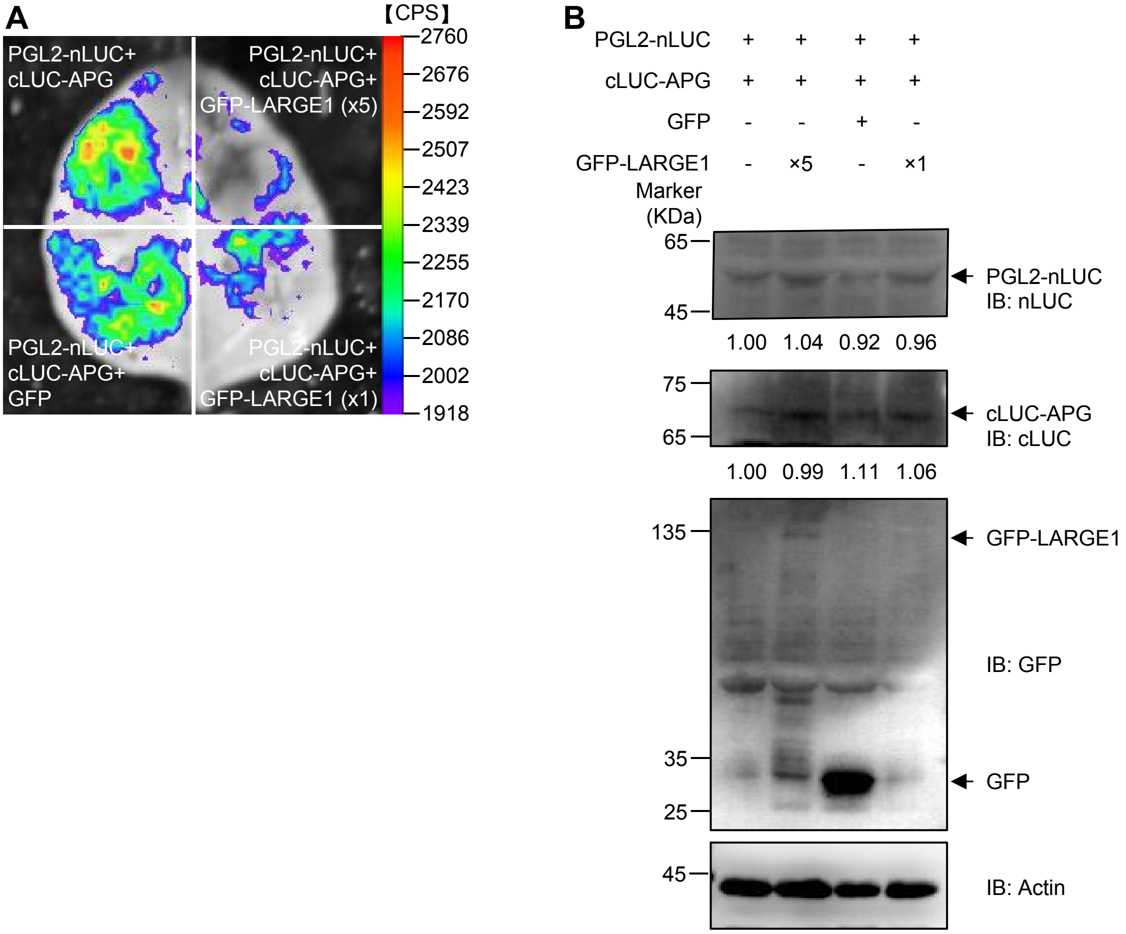

Supplementary Figure 6. LARGE1 competitively inhibits APG-PGL2 binding

(A) The Split-luciferase complementation assay reveals GFP-LARGE1-mediated inhibition of PGL2-nLUC/cLUC-APG complex formation. Transient co-expression assays were performed by infiltrating *Nicotiana benthamiana* leaves with *Agrobacterium* cultures carrying 35S promoter-driven constructs (*PGL2-nLUC*, *cLUC-APG*, *GFP*, or *GFP-LARGE1*) in designated combinations. Luminescence signals were detected using D-luciferin as luciferase substrate.

(B) Immunoblot analysis protein expression profiles across experimental groups in panel A. Protein lysates were immunoprobed using epitope-specific antibodies against nLUC, cLUC, GFP and Actin (endogenous control).

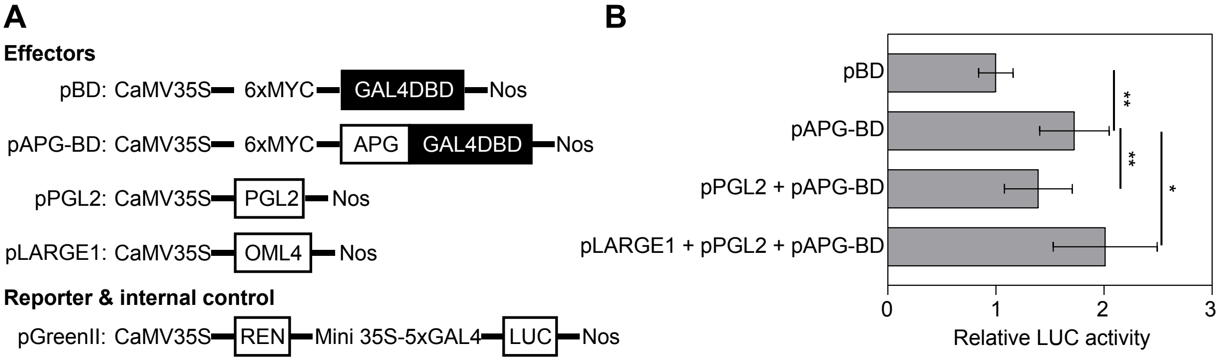

Supplementary Figure 7. APG transcriptioanal activation activity is affected by PGL2 and LARGE1

(A) Effector, reporter and internal control constructs used in the dual-luciferase reporter (DLR) assay system in *N. Benthamina*. The reporter gene *LUC* and internal control *REN* are in the same vector.

(B) Transcriptional activation ability of APG as revealed by relative LUC activity of the corresponding reporters listed in (A). pBD is used as negative control.

Values are given as means ± SD (n=24). GAL4DBD is short for GAL4 DNA binding domain. 5 × GAL4, five copies of the GAL4 binding element. LUC, the firefly luciferase gene; REN, the Renilla luciferase gene. **, $P < 0.01$; *, $P < 0.05$.

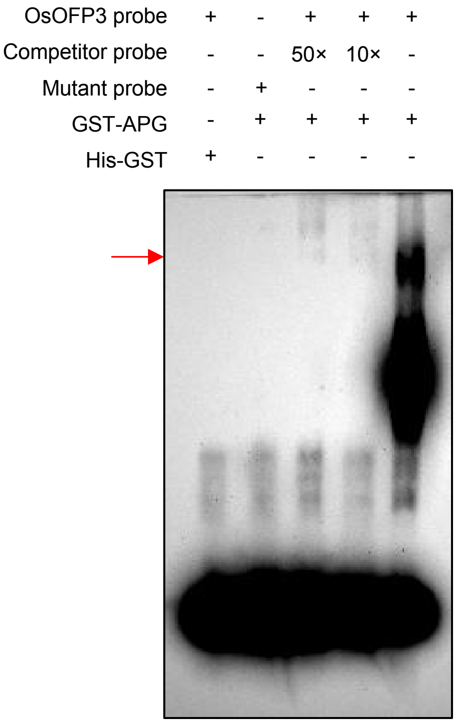

Supplementary Figure 8. EMSA assay of binding between APG and the predicted potential target gene *OsOFP3*

APG binds to *OsOFP3* promoter. The competitor probe was added at 10- and 50-fold more than the labeled probes respectively. The red arrow indicates the position of the shifted bands.

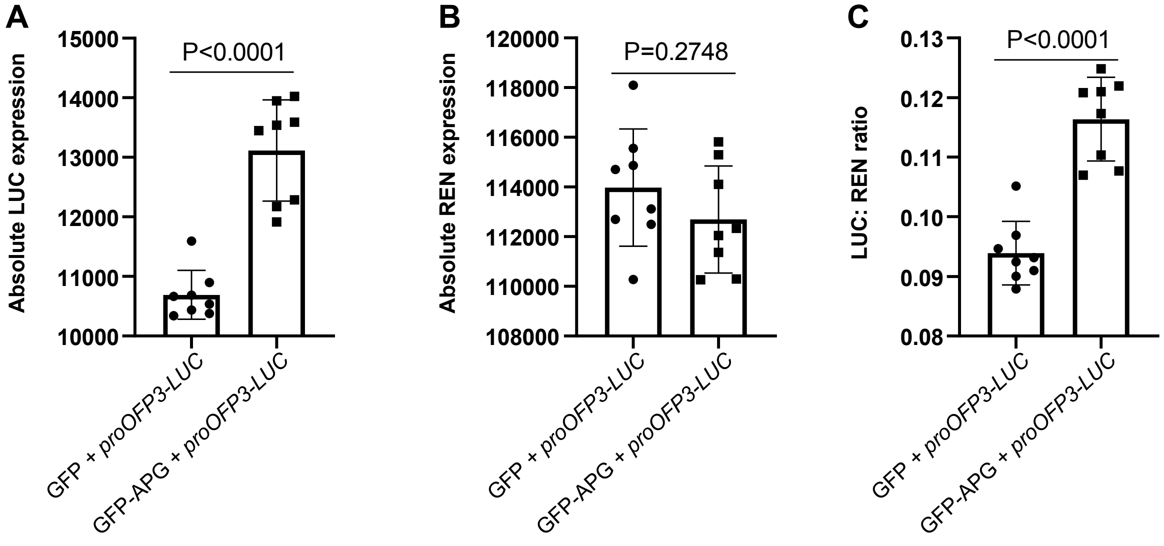

Supplementary Figure 9. Dual-luciferase reporter gene assay validates that APG can enhance the promoter activity of *OFP3*
Absolute and relative LUC and REN expression levels from the pGreenII 0800-LUC construct following 2 days infiltration into *N. Benthamina* leaves. A, Absolute LUC activity. B, Absolute REN activity. C, LUC to REN ratio.

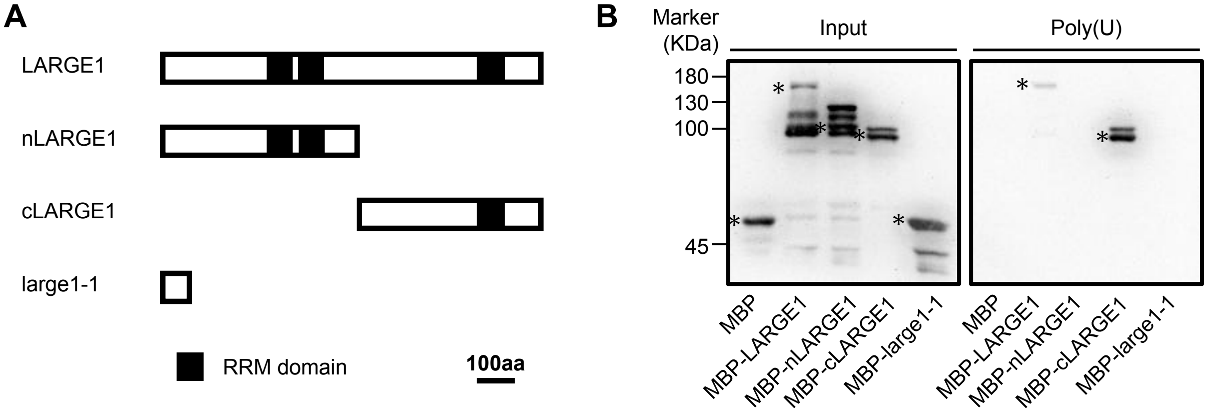

Supplementary Figure 10. RNA binding activity of LARGE1 *in vitro* assay.

(A) Schematic structure of LARGE1, nLARGE1, cLARGE1 and mutated protein encoded by *large1-1*.

(B) LARGE1 binds RNA *in vitro*. Purified fusion proteins were incubated with poly (U) agarose beads. Asterisks indicate the target proteins immunodetected with anti-MBP antibody.

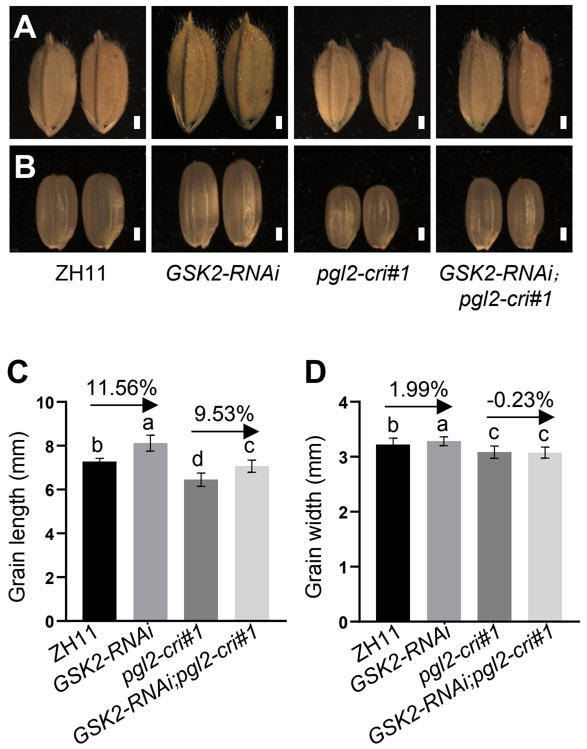

Supplementary Figure 11. *PGL2* acts genetically with *GSK2*.

(A) and (B) Morphological characteristics of grains from ZH11, *GSK2-RNAi*, *pgl2-cri#1*, and *GSK2-RNAi;pgl2-cri#1* plant lines. (C) and (D) Statistical analysis of grain length and width in ZH11, *GSK2-RNAi*, *pgl2-cri#1*, and *GSK2-RNAi;pgl2-cri#1*. Data are presented as mean values ± SD, with a sample size of at least 154 grains ($n \geq 154$). Columns marked with distinct lowercase letters indicate statistically significant differences at the $P < 0.01$ level, as determined by one-way analysis of variance (ANOVA). Scale bars: 1 mm (applicable to panels C and D).

Supplementary Table 1. Primers used in this study

**Primers for real-time PCR**	
Actin-F	5'-TGCTATGTACGTCGCCATCCAG-3' (Lyu, Wang et al. 2020)
Actin-R	5'-AATGAGTAACCACGCTCCGTCA-3' (Lyu, Wang et al. 2020)
PGL2-F	5'-GCAGAAGGTCGTCGCGTGGCTCCATC-3' (Jang, An et al. 2017)
PGL2-R	5'-GCGGAGGATGCTGCGGATGATCTCCG-3' (Jang, An et al. 2017)
**Primers for constructs in overexpression of PGL2**	
PGL2-OE-F	5'-GCAGGAATTCAAGCTTATGTCGAGCAGAAGGTCGTCG-3'
PGL2-OE-R	5'-AGTCACTATGGTCGACTCAGGAGCGGAGGATGCTGC-3'
**Primers for constructs in yeast two hybrid assays**	
LARGE1-pPR3N-F	5'-AGATTACGCTGGATCCATGCCATCTCAGGTCATGGA-3'
LARGE1-pPR3N-R	5'-ACCACTGCTTGGATCCTCAGTCCTTGGCAGGCCCTGT-3'
LARGE1-pGADT7-F	5'-ATGGAGGCCAGTGAATTCATGCCATCTCAGGTCATGGA-3'
LARGE1-pGADT7-R	5'-CATCTGCAGCTCGAGCTCGTCCTTGGCAGGCCCTGTAG-3'
PGL2-pDHB1-F	5'-GGCCAGGCCTCCATGGATGTCGAGCAGAAGGTCGTCGCG-3'
PGL2-pDHB1-F	5'-CCAAGATATACCATGGGGAGCGGAGGATGCTGCGGATG-3'
PGL2-pGADT7-F	5'-ATGGAGGCCAGTGAATTCATGTCGAGCAGAAGGTCGTCGCG-3'
PGL2-pGADT7-R	5'-CATCTGCAGCTCGAGCTCGGAGCGGAGGATGCTGCGGATG-3'
APG-pGBKT7-F	5'-CGAATTCCCGGGGATCCGTATGCTACGCGGGAACGACACCGGC-3'
APG-pGBKT7-F	5'-TGCAGGTCGACGGATCCCCGCCTGCTTCACGGCGGGGGCGTT-3'
**Primers for constructs in luciferase assays**	
PGL2-nLuc-F	5'-GAGCTCGGTACCCGGGGATCCATGTCGAGCAGAAGGTCGTCGCG-3'
PGL2-nLuc-R	5'-GCGTACGAGATCTGGTCGACGGAGCGGAGGATGCTGCGGATG-3'
cLUC-OML4-F	5'-GGGGCGGTACCCGGGGATCCATGCCATCTCAGGTCATGGA-3'
cLUC-OML4-R	5'-CGAAAGCTCTGCAGGTCGACTCAGTCCTTGGCAGGCCCTGT-3'
cLUC-APG-F	5'-GGGGCGGTACCCGGGGATCCATGCTACGCGGGAACGACACCGGC-3'
cLUC-APG-R	5'-CGAAAGCTCTGCAGGTCGACTCACGCCTGCTTCACGGCGGGGGC-3'
**Primers for constructs in BiFC assays**	
LARGE1-cYFP-F1	5'-CACGGGGGACTCTAGAATGGCCGACAAGCAGAAGAA-3'
LARGE1-cYFP-R1	5'-TCCATGACCTGAGATGGCATCGCATAGTCAGGAACATC -3'
LARGE1-cYFP-F2	5'-GATGTTCCTGACTATGCGATGCCATCTCAGGTCATGGA-3'
LARGE1-cYFP-R2	5'-AGTCACTATGGTCGACTCAGTCCTTGGCAGGCCC-3'
PGL2-nYFP-F1	5'-CACGGGGGACTCTAGAATGGCCGACAAGCAGAAGAA-3'
PGL2-nYFP-R1	5'-ACGACCTTCTGCTCGACATCGCATAGTCAGGAACATC-3'
PGL2-nYFP-F2	5'-GATGTTCCTGACTATGCGATGTCGAGCAGAAGGTCGT-3'
PGL2-nYFP-R2	5'-AGTCACTATGGTCGACTCAGGAGCGGAGGATGC-3'
**Primers for constructs in pull-down assays**	
FLAG-LARGE1-F	5'-GTTCCAGATTACGCTGGATCCGAATTCCCATCTCAGGTCATGGATCA-3'
FLAG-LARGE1-R	5'-AGTGGTGGTGGTGGTGGTGCTCGAGGTCCTTGGCAGGCCCTGTAG-3'
MBP-LARGE1-F	5'-GGATTTCAGAATTCGGATCCATGCCATCTCAGGTCATG-3'
MBP-LARGE1-F	5'-GGCCAGTGCCAAGCTTGCTTTCAGTCCTTGGCAGGCCC-3'
GST-PGL2-F	5'-ATCTGGTTCCGCGTGGATCCATGTCGAGCAGAAGGTCGTCG-3'
GST-PGL2-R	5'-AGTCAGTCACGATGCGGCCGCTCTCAGGAGCGGAGGATGCTGC-3'
FLAG-APG-F	5'-GTTCCAGATTACGCTGGATCCGAATTCCTACGCGGGAACGACACCGGC-3'
FLAG-APG-R	5'-AGTGGTGGTGGTGGTGGTGCTCGAGCGCCTGCTTCACGGCGGGGGC-3'

**Primers for constructs in Co-IP assays**	
GFP-LARGE1-F	5’-TGAACTATACAAAGGCGCGCCAATGCCATCTCAGGTCATGGA-3’
GFP-LARGE1-R	5’-CTCTAGAACTAGTTAATTAATCAGTCCTTGGCAGGCCCTGT-3’
MYC-PGL2-F	5’-ACTTGAATTCGGTACCCATGTCGAGCAGAAGGTCGT-3’
MYC-PGL2-R	5’-TAGGCTACGTAGGATCCATCAGGAGCGGAGGATGC-3’
**Primers for constructs in transcription activity assays**	
GAL4DBD-APG-F	5’-AAGCTTATCGATACCGTCGACATGCTACGCGGGAACGACACC-3’
GAL4DBD-APG-R	5’-GATTTTTGCGGACTGGGTACCTCACGCCTGCTTCACGGCGGG-3’
pRT107-PGL2-F	5’-AAGCTTATCGATACCGTCGACATGTCGAGCAGAAGGTCGTCG-3’
PRT107-PGL2-R	5’-GATTTTTGCGGACTGGGTACCTCAGGAGCGGAGGATGCTGCG-3’
pRT107-LARGE1-F	5’-AAGCTTATCGATACCGTCGACATGCCATCTCAGGTCATGGAT-3’
pRT107-LARGE1-R	5’-GATTTTTGCGGACTGGGTACCTCAGTCCTTGGCAGGCCCTGT-3’
MYC-BD-F	5’-ACTTGAATTCGGTACCCATGAAGCTACTGTCTTCTATCGAA-3’
MYC-BD-R	5’-TAGGCTACGTAGGATCCACGGCGATACAGTCAACTGTC-3’
MYC-APG-BD-F	5’-ACTTGAATTCGGTACCCATGCTACGCGGGAACGACAC-3’
APG-BD-R	5’-TTCGATAGAAGACAGTAGCTTCATCGCCTGCTTCACGGCGGGGGCGTT-3’
APG-BD-F	5’-AACGCCCCCGCCGTGAAGCAGGCGATGAAGCTACTGTCTTCTATCGAA-3’
MYC-APG-BD-R	5’-TAGGCTACGTAGGATCCACGGCGATACAGTCAACTGTC-3’
**Primers for constructs in EMSA assays**	
OFP3-EMSA-F	5’-TTTGTTATCCACGTGCACGCAATATCACGAGCCACCCCCC-3’
OFP3-EMSA-R	5’-GGGGGGTGGCTCGTGATATTGCGTGCACGTGGATAACAAA-3’
OFP3-Biotin-F	5’-TTTGTTATCCACGTGCACGCAATATCACGAGCCACCCCCC-3’
OFP3-Biotin-R	5’-GGGGGGTGGCTCGTGATATTGCGTGCACGTGGATAACAAA-3’
OFP3-Mutant probe -F	5’-TTTGTTATCCCGCGACACGCAATATCCGCGACCACCCCCC-3’
OFP3-Mutant probe -R	5’-GGGGGGTGGTCGCGGATATTGCGTGTCGCGGGATAACAAA-3’
**Primers for constructs in *OFP3* promoter activity assays**	
pGreenII 0800-OFP3-LUC-F	5’-GCAGCCCGGGGGATCCGAGGGGTAATGCGCGCCCTC-3’
pGreenII 0800-OFP3-LUC-R	5’-TTGGCGTCTTCCATGGGGTCCCCATGACGCTAGTGT-3’
